# Dating the Colonisation and Source of Adaptive Genetic Variation in the Iago Sparrow (*Passer iagoensis*)

**DOI:** 10.1111/mec.70587

**Published:** 2026-11-03

**Authors:** José Cerca, Ângela M. Ribeiro, Michael Matschiner, Rosa Alicia Jiménez, Hugo Pereira, Zheng Oong, Gabriel David, Per Unneberg, Yan Wong, Gabriele Maria Sgarlata, Pim Edelaar, Tomáš Albrecht, Glenn‐Peter Saetre, Pedro Cardia, Augusto Faustino, Evandro P. Lopes, Rauri C. K. Bowie, Mark Ravinet, Martim Melo

**Affiliations:** ^1^ Section for Ecology, Biodiversity and Evolution (EBE), Department of Biosciences University of Oslo Oslo Norway; ^2^ Department of Bioinformatics and Genetics Swedish Museum of Natural History Stockholm Sweden; ^3^ SciLifeLab Karolinska Institutet Science Park Solna Sweden; ^4^ CISAS—Center for Research and Development in Agrifood Systems and Sustainability Polytechnic University of Viana do Castelo Viana do Castelo Portugal; ^5^ Natural History Museum University of Oslo Oslo Norway; ^6^ Faculty of Biology Ludwig Maximilian University Munich Planegg‐Martinsried Germany; ^7^ Museum of Vertebrate Zoology University of California Berkeley California USA; ^8^ Department of Integrative Biology University of California Berkeley California USA; ^9^ Escuela de Biología Universidad de San Carlos de Guatemala Ciudad de Guatemala Guatemala; ^10^ CIBIO, Centro de Investigação em Biodiversidade e Recursos Genéticos, InBio, Laboratório Associado Universidade do Porto Vairão Portugal; ^11^ Department of Animal Behaviour Bielefeld University Bielefeld Germany; ^12^ Bell Museum and Department of Ecology, Evolution and Behavior University of Minnesota Saint Paul Minnesota USA; ^13^ Zooekologi, Department of Ecology and Genetics Uppsala University Uppsala Sweden; ^14^ Department of Cell and Molecular Biology, National Bioinformatics Infrastructure Sweden, Science for Life Laboratory Uppsala University Uppsala Sweden; ^15^ Big Data Institute, Li Ka Shing Centre for Health Information and Discovery University of Oxford Oxford UK; ^16^ Department of Evolution and Ecology University of California Davis California USA; ^17^ Department of Molecular Biology and Biochemical Engineering University Pablo de Olavide Seville Spain; ^18^ Department of Biology Lund University Lund Sweden; ^19^ Institute of Vertebrate Biology Czech Academy of Sciences Brno Czechia; ^20^ Department of Zoology, Faculty of Science Charles University Praha Czechia; ^21^ STRIX – Innovation for Global Biodiversity Alagoa Office & Retail Center Carcavelos Portugal; ^22^ ICBAS, Instituto de Ciência Biomédicas Abel Salazar Universidade do Porto Porto Portugal; ^23^ Instituto de Engenharia e Ciências do Mar Universidade Técnica do Atlântico Mindelo Cabo Verde; ^24^ School of Life Sciences University of Nottingham Nottingham UK; ^25^ MHNC‐UP Natural History and Science Museum of the University of Porto Porto Portugal; ^26^ FitzPatrick Institute of African Ornithology University of Cape Town Rondebosch South Africa

## Abstract

Ancestral adaptive variation is thought to play an important role in facilitating adaptation to insular environments. However, to properly evaluate its role, we need to determine the age of genetic variation. In this study, we investigate the Iago sparrow ( *Passer iagoensis* ), a generalist endemic to the Cabo Verde archipelago, to understand how genetic diversity is maintained in an island lineage where populations are likely ephemeral and adaptation to environmental extremes is ongoing. Using whole‐genome sequencing and phenotypic data, we inferred the species' evolutionary history, population structure, and signatures of selection. Our results show that the Iago sparrow diverged from its closest mainland relative approximately 4.26 million years ago, although the colonisation likely occurred only around 2 million years ago. Population structure analyses reveal three distinct lineages, corresponding to geographic divisions within the archipelago, with the Rombos islets population exhibiting significant phenotypic divergence, particularly in bill morphology. Genome scans and phenotypic measurements identified signatures of divergent selection in genes associated with bill size (*HMG20B*) and bill shape (*ALX1*), suggesting adaptive evolution in response to local environmental conditions. Our ancestral recombination graph analysis provided temporal estimates for adaptive variation associated with these genes, suggesting that adaptive haplotypes observed in the Rombos population arose after colonisation of the archipelago. This contrasts with patterns described in Darwin's finches, where ancestral variation likely originated prior to colonisation. Our findings highlight the interplay of genetic drift, gene flow, and selection in shaping island species' genetic diversity. The Iago sparrow, a generalist thriving across diverse habitats, provides insights into the mechanisms that maintain genetic diversity in island lineages despite small population sizes and pronounced environmental heterogeneity.

## 1 Introduction

Organisms inhabiting remote archipelagos often undergo remarkable diversification, reflected not only in species numbers but also in phenotypic diversity, as seen in Darwin's finches and Caribbean anoles (Losos and Ricklefs 2009). This presents a fascinating evolutionary paradox (Cerca et al. 2023; Stuessy et al. 2014): On the one hand, there are often striking levels of phenotypic and ecological variation, while on the other, there are multiple factors that reduce genetic diversity and should constrain diversification. Colonisation of an archipelago, or of individual islands within an archipelago, typically involves founder events that diminish genetic diversity. Moreover, the restricted geographic range of island populations results in smaller population sizes (Arnold et al. 2008; Frankham 2008; Wang et al. 2014; White and Searle 2007; Leroy et al. 2021) and greater susceptibility to frequent local extinctions caused by natural disturbances such as volcanic eruptions and associated tsunamis (Vandergast et al. 2004; Carson et al. 1990; Carson and Johnson 1975; Ramalho et al. 2015; Castilla‐Beltrán et al. 2021). These iconic radiations therefore offer a unique opportunity to explore how and when genetic variation arises, is maintained, and contributes to lineage diversification (Cerca et al. 2023).

One proposed hypothesis to explain the high levels of phenotypic and ecological niche differentiation observed on islands is that a substantial portion of ancestral variation is preserved within the colonising lineages (Lamichhaney et al. 2015; Cerca et al. 2023). However, the role that ancestral variation plays in driving adaptation and divergence is likely complicated by the ephemeral nature of insular populations. Island environments are vulnerable to geological and climatic disturbances, and the populations they sustain are small and fragmented. Population bottlenecks are therefore expected not only at the time of initial colonisation but also following the colonisation of additional islands and the repeated re‐establishment of populations after catastrophic events (Clegg et al. 2002). This means that across insular systems, genetic variation is more likely to be lost and coalescent times within populations will be shorter, on average.

Nonetheless, there are several well‐known examples of island divergence where ancestral alleles can contribute to adaptation. For example, in Darwin's finches, alleles associated with bill size and shape have been characterised as ancestral variation, as the divergence of these adaptive alleles predates estimated speciation events (Lamichhaney et al. 2015; Han et al. 2017; Rubin et al. 2022). However, whether this ancestral variation predates the colonisation of the Galápagos remains an open question. Similarly, in the Hawaiian radiation of *Metrosideros* plants, genetic variation appears to be older than the radiation and possibly predates island colonisation (Choi et al. 2021). Both of these examples illustrate that our current understanding of the role of ancestral variation in island adaptation comes from radiations with considerable and clear phenotypic diversity. Whether ancient allelic diversity contributes more broadly to insular adaptation remains unclear, and more studies on the evolutionary dynamics of island populations are needed to fully address this question.

In 1831, the HMS Beagle set sail with the young naturalist Charles R. Darwin on board. While his Galápagos collections have transformed our understanding of evolution, other aspects of the journey remain less explored. The HMS Beagle's first stop was Santiago Island (‘Saint Iago’ Island) in the Cabo Verde archipelago. Among the species Darwin collected were two *Passer* sparrow species: the Spanish sparrow ( *Passer hispaniolensis* ) and a species new to science, later described by J. Gould as *Passer iagoensis* and endemic to the archipelago (the Iago or Cape Verde Sparrow; Figure 1A). The Iago sparrow demonstrates an extraordinary ability to thrive across the diverse environments of the archipelago, which range from arid regions with precipitation as low as 80 mm of annual precipitation to mesic areas receiving up to 1600 mm per year (Barone and Delgado 2011; Summers‐Smith 1984). This ecological flexibility further extends to elevation, with populations occurring from sea level to 1500 m a.s.l., encompassing a broad spectrum of climatic and ecological conditions. The Iago sparrow inhabits nine out of the 10 islands and three of the four main islets of the archipelago. Moreover, the species is common in human‐altered environments, including urban areas, where it coexists with the Spanish and, to a lesser extent, house sparrows ( *Passer domesticus* ). However, unlike Darwin's Finches, the Iago sparrow has not undergone obvious ecological specialisation or adaptive radiation and is recognised as a monotypic species (Christidis et al. 2025). Few studies have focused on this species, and most of what is published is observational (Summers‐Smith 1984). Previous mitochondrial analyses revealed no clear genetic structuring (Päckert et al. 2021). Beyond this, however, there is virtually no understanding of the genomic or phenotypic variation that may be crucial for the ability of this species to thrive across such diverse environments. Considering the species broad geographic distribution and remarkable ecological flexibility, investigating the evolutionary history of Iago sparrow may offer valuable insights into the role of ancestral adaptive variation in shaping the diversity of insular biological systems.

**FIGURE 1:**
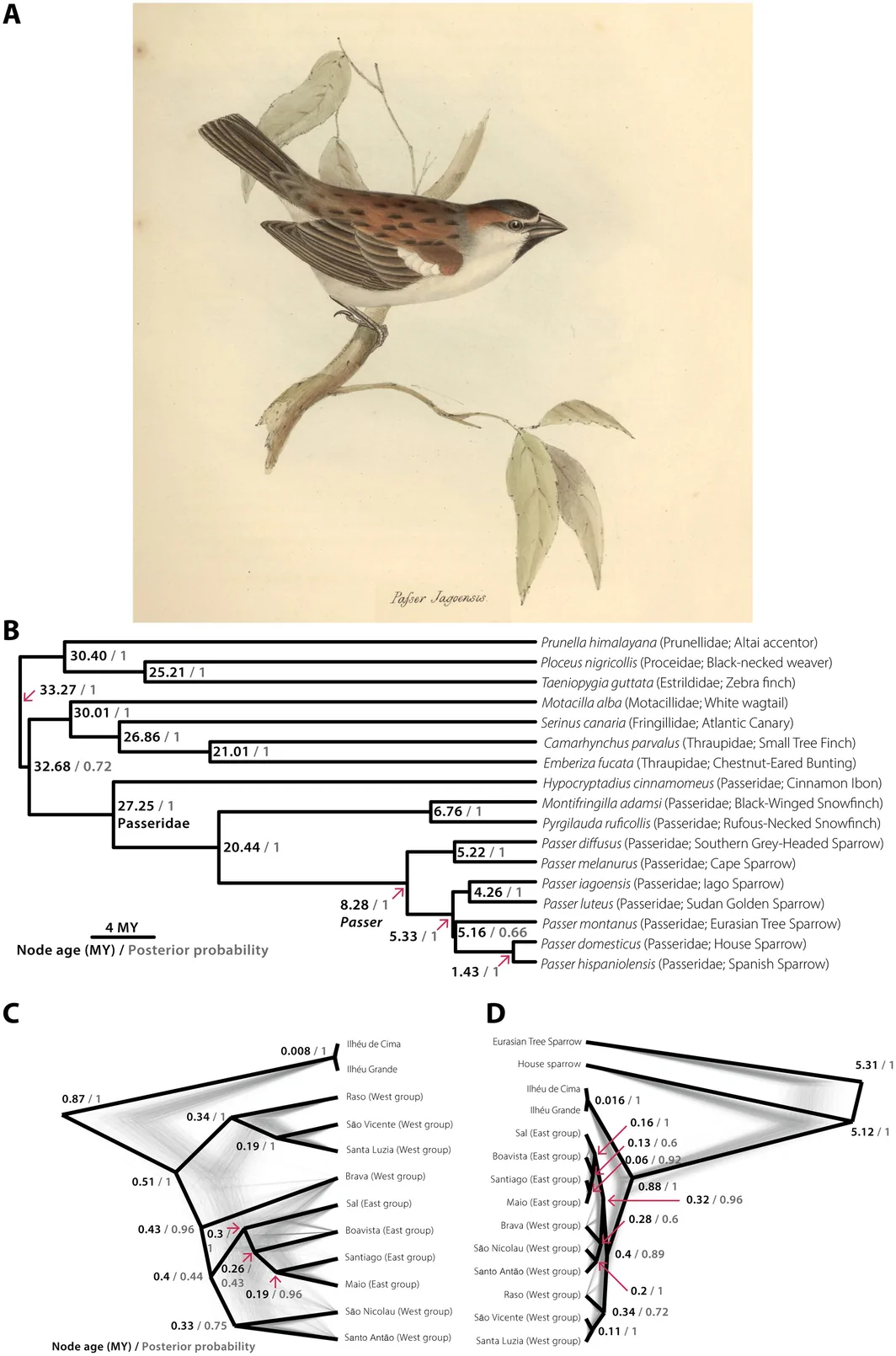
Species and population‐level relationship. (A) John Gould's drawing published in The Zoology of the Voyage of H.M.S. Beagle (part 3; public domain; likely illustrated by John's wife, Elizabeth Gould); (B) *StarBeast2* species tree for 17 species (seven avian families within the order Passeriformes) obtained with whole‐genome data. The lineages Passeridae and *Passer* are labelled on the nodes for convenience. (C) *SNAPP* cloudogram of the ddRADseq data including only Iago sparrow populations. (D) *SNAPP* cloudogram of the whole‐genome resequencing data including Iago sparrow populations and two outgroups (the house and Eurasian tree sparrows). Node ages and posterior probabilities are provided in each tree and colour‐coded in black and grey, respectively.

Here, we set out to determine when and how genetic variation arose in the Iago sparrow as it colonised and established across the Cabo Verde archipelago. To achieve this, we first estimated (1) when the species diverged from its closest relatives, thereby providing an upper limit on the timing of archipelago colonisation. To do this, we inferred the phylogenetic relationship of the Iago sparrow and its divergence time relative to other *Passer* sparrows using a time‐calibrated whole‐genome phylogeny. We then asked (2) whether this endemic species harbours genetic variation associated with biogeographical variation across the archipelago, using population structure and landscape genomic analyses. Finding high levels of divergence within the species, we next used phenotypic and genomic data to (3) identify signatures of selection driving adaptation in the Iago sparrow. Finally, (4) we estimated the age of allelic variation across the genome to test whether variation linked to adaptation arose prior to or following the colonisation of the archipelago. Our main aim with this work is to improve our understanding of the evolutionary dynamics of genetic variation in a generalist island system and to provide an additional empirical case to assess the broader question of whether ancient variation plays a wider role in adaptation or is limited to a handful of special systems.

## 2 Methods

### 2.1 Study System and Data Collection

The Cabo Verde archipelago comprises 10 volcanic islands and four islets situated approximately 600 km off the coast of mainland West Africa (Romeiras and Duarte 2019). Despite its location within the Sahelian arid climatic zone, the archipelago exhibits a mosaic of diverse environments owing to the dramatic topography of some islands. These volcanic islands are arranged in a horseshoe shape, where the oldest and flattest islands (Sal, Boavista and Maio) are closer to the mainland. The Iago Sparrow is a permanent resident of all islands and islets, except on the islet of Branco and on Fogo Island, where its occurrence is irregular and unconfirmed, respectively. Within these islands, the species inhabits a wide range of environmental conditions. For instance, on Brava, Santo Antão and São Nicolau (western), as well as on Santiago (eastern), we sampled birds inhabiting both arid and humid subtropical areas. During our expedition in September and October 2014, we surveyed Branco and Fogo but detected no Iago Sparrows. Our sampling scheme was designed so that: (i) sampling points covered the entire species range, from small islets to larger islands; and (ii) location and number of sampling points encompassed within‐island environmental variability. In September and October 2014, we captured a total of 415 birds across the entire species' range (9 islands and 3 islets; 26 localities) using mist‐ or hand‐held nets. Upon capture, birds were measured and a blood sample obtained (20–30 μL collected from the brachial wing vein and stored in RNAlater). Birds were released at point of capture.

### 2.2 Environmental Context

Climatic data (WorldClim version 2.1; 19 bioclimatic variables, 30 arc‐seconds resolution) describing precipitation and temperature (Fick and Hijmans 2017) were used to study the climatic conditions across the range of the species within the Cabo Verde archipelago. The 30 arc‐second dataset is the highest resolution possible with WorldClim and is roughly approximate to a 1 km resolution at the equator. Therefore this resolution is sufficient to capture climate variation across Cabo Verde where major island sizes range from 35 to 991 km^2^. A Principal Component Analysis based on the 19 bioclimatic variables extracted for each occurrence was used to reduce the dimensionality of the data and summarise the climatic envelope of the species. We used Bio12 (annual precipitation; mm) as a proxy to classify sites into three groups: hyper arid: Bio12 < 100; arid: 100 < Bio12 < 250; mesic: Bio12 > 250. All analyses were performed in R v4.2.3 (R Core Team 2023; packages: nlme).

### 2.3 Phenotyping

We measured five morphological variables for 415 birds: wing‐length, tarsus‐length, bill‐length, bill‐depth and bill‐width. Wing‐length was measured from the carpal joint to the tip of the longest primary feather using a wing‐rule to an accuracy of 0.5 mm. Tarsus‐length and bill measurements were obtained with a digital calliper to an accuracy of 0.1 mm. Tarsus‐length was measured from the middle of the intertarsal joint to the distal edge of the last scale before the toes (Pyle 1997). Bill‐length was measured as the distance from the last crown feathers to the distal tip of the upper mandible (exposed culmen); bill‐depth and bill‐width were measured at the anterior‐end of the nares. Martim Melo collected all measurements following a pilot study on repeatability (same operator consistently obtains the most similar measurements on the same subject) that suggested that he was the most reliable operator. Besides the morphological data collected in the field, we also derived a composite metric—bill volume—with the rationale that it might play a role in thermoregulation (Greenberg et al. 2012; Tattersall et al. 2017; Slatkin 1984). We calculated bill volume using the formula for the volume of an ellipsoid cone: V = 1/3pi*r*R*h; hence Bill Volume = 1/3*pi *(1/2bill‐width × 1/2bill‐depth × bill‐length).

Before conducting morphological analyses, we excluded all immature birds, resulting in a dataset of 375 adult individuals. To meet the assumption of normality, we log10‐transformed the data following an assessment using QQ‐plots and Shapiro's test.

We aimed to evaluate the roles of geography (using island identity as a proxy), genetics (genetic clusters), and climate in shaping morphological variation in six traits. First, we conducted a two‐way ANOVA to assess whether morphology was influenced by sex and geography. Given the presence of sexual dimorphism (see Results section), we tested whether the dimorphism varied across the species' range by including an interaction term (island × sex). Second, we examined the effect of genetic structure by grouping the data into the three clusters identified (see Results section). The ANOVA, followed by a post hoc Tukey's HSD test, allowed us to determine which groups occupied significantly different regions of the morphospace. Finally, we investigated the influence of climate (summarised by two principal components, PC1 and PC2) on morphology. This analysis was conducted using a MANOVA, followed by ANOVA to explore significant results.

Given the potential role of bill size and the observation that birds from Rombos exhibited overall larger bills, we further tested whether this trait was significantly larger in the Rombos population compared to Brava, a geographically close island (7 km) with contrasting environmental conditions (Brava: mesic; Rombos: hyper‐arid). We used *t*‐tests for this comparison, and *p*‐values were adjusted for multiple comparisons using the Holm method (Holm 1979). All analyses were performed in R v4.2.3 (R Core Team 2023; packages: nlme).

To formally test for the effects of divergent selection on bill volume, tarsus‐length, and wing‐length, we estimated Pst values. Pst is the phenotypic analog of Fst (Weir and Cockerham 1984) that measures population differentiation relative to the total variance among populations (Brommer 2011; Pst = c *σ* ^2^b/c *σ* ^2^b + 2 h^2^ *σ* ^2^w). The variance components between (*σ* ^2^b) and within (*σ* ^2^w) populations were estimated after fitting a mixed‐effects model, with population as a fixed effect and sex as a random effect. For the trait‐specific heritability (h^2^) values, we used previous estimates for House sparrows (h^2^ tarsus = 0.48, Jensen et al. 2003; h^2^ wing = 0.41, Jensen et al. 2003, h^2^ bill depth and length = 0.38, Lundregan et al. 2018). For the coefficient *c*, the additive genetic proportion of phenotypic differences between populations, (range 0–1), we tested two values: *c =* 1 meaning that all the phenotypic variation between populations is determined by genetic variation, and *c* = 0.5 meaning that the environment is causing part of the phenotypic variance between the populations. We estimated 95% confidence intervals (CIs) for Pst using a non‐parametric bootstrapping approach. Bootstrapping was performed by resampling (999 iterations) the dataset with replacement within each population. To provide insights into the degree of differentiation caused by selective versus neutral processes (Brommer 2011), we compared Pst to differentiation of genetic variation (Fst), with the rationale that Fst estimates obtained from neutral genetic variation reflect the divergence produced by genetic drift alone (Merilä and Crnokrak 2001; Edelaar and Björklund 2011; Edelaar et al. 2011). Thus, if environmental pressures impose selection in phenotype (morphological traits), we anticipate Pst values higher than Fst, suggesting that genetic drift is not the main force driving phenotypic differentiation and that selection may be strong enough to counteract the homogenising effects of gene flow. Alternatively, if there is no departure from neutral expectation, then Pst ≈ Fst. Pst was considered significantly different from Fst when the respective confidence intervals did not overlap. Pst estimation in wild populations has some caveats: the components of additive genetic variance within (h^2^) and among populations (C) can only be assumed and not tested. F_ST_ should also be estimated only on bi‐allelic loci, which we achieve here using estimates from SNPs. However, we consider the Pst‐Fst comparison to be a valuable tool for generating hypotheses on selection, which can be further explored. All analyses were performed in R v4.2.3 (R Core Team 2023; packages: nlme).

### 2.4 Molecular DNA & Sequencing

DNA from Iago sparrows was extracted from blood samples using the JETQUICK Tissue DNA Spin Kit (Genomed, Germany) following the manufacturer's protocol. For all other *Passer* species, DNA was extracted using a Qiagen Blood & Tissue DNEasy kit (Qiagen, California, USA). The presence and quality of DNA were verified by electrophoresis on a 0.8% agarose gel, and DNA concentration (ng/μL) was quantified using a Qubit 2.0 Fluorometer (Life Technologies, ThermoFisher Scientific, USA). Sequencing was performed using two different strategies for the two different genomic datasets. Firstly, for our *Passer* phylogenomic analysis, all *Passer* species ( *P. diffusus* , *P. melanurus* , *P. iagoensis* , *P. luteus* , *P. montanus* , *P. domesticus* , and *P. hispaniolensis; n* = 7 in total) were prepared using an Illumina TruSeq gDNA 180 bp kit and sequenced to a minimum of 30× coverage on an Illumina Hi‐Seq X at Genome Quebec, McGill University, Canada. The purpose of this level of coverage was to ensure more accurate base‐calling as a basis for phylogenetic reconstruction.

All population level Iago sparrow samples (*n* = 43) were prepared for sequencing using a NexteraPE kit and sequenced at the Norwegian Sequencing Centre at the University of Oslo, to a target coverage of 10×. The aim of this dataset was to provide a broad‐scale survey of genomic variation across all islands in the archipelago while generating population level genomic data for the Rombos population to support high‐resolution genome scan analyses. See Table S1 for a full breakdown of sequenced individuals.

To provide a more comprehensive population genomic dataset, balancing genome coverage with the number of individuals, we also applied a double‐digest restriction‐site‐associated DNA sequencing (ddRAD‐seq) approach for a total of 224 individuals from across the archipelago. This allowed us to examine population structure among islands in greater detail as well as to estimate migration rates and clustering. We followed the protocol described by Peterson et al. (2012). Briefly, 500 ng of genomic DNA were digested over 3 h using the restriction enzymes EcoRI and NlaIII (New England Biolabs, USA). After digestion and bead clean‐up, each sample was labelled by the ligation of one of 24 different adapters. Adapter ligation was followed by pooling sets of up to 24 individual samples (10 pools of 24, one pool of 21, one pool of 17, two pools of 16, one pool of 15 and one pool of 14 resulting in 16 pools with a total of 224 samples) and two consecutive bead clean‐ups. The cleaned product was PCR‐amplified and indexed using the Phusion DNA polymerase (New England Biolabs, USA). After bead clean‐up and qPCR, the 16 pools were combined in an equimolar manner. The final library was run on one lane of the Illumina NovaSeq SP to generate 150 bp paired‐end sequence reads. The sequencing was carried out at the QB3 Vincent J. Coates Genomic Sequencing Laboratory at UC Berkeley, in an Illumina NovaSeq 6000 machine.

### 2.5 Bioinformatics Processing of RADseq Data

*Illumina* read files underwent quality check using FASTQC (Andrews 2017). We started by using process radtags from Stacks v2.54 (Rochette et al. 2019) to demultiplex our data, specifying: the enzymes used (nlaIII and ecoRI), discard reads with low quality scores (−q), rescue barcodes and RAD‐Tag cut sites (−r), clean data, remove any read with an uncalled base (−c) and a barcode distance of 1 (−barcode‐dist‐1). We then aligned the reads to the reference genome of the House sparrow (Elgvin et al. 2017) using bwa‐mem (Li and Durbin 2009). We used Stacks v2.54 (gstacks), to generate RAD loci and populations (Rochette et al. 2019). For the latter, we specified 80% as the minimum percentage of individuals across populations required to process a locus (−R), and output the data as a variant call format file (vcf) and fasta file (sequences).

### 2.6 Bioinformatics Processing of the Whole‐Genome Data

The *Illumina* read files underwent quality check using FASTQC 0.11.9 (Andrews 2017), and regions containing illumina‐adapters or with low‐quality sequencing reads were removed using *Trimmomatic* 0.36 (Bolger et al. 2014). The remaining reads were then aligned to the reference genome of the House Sparrow (Elgvin et al. 2017) using bwa‐mem 0.7.17 (Li and Durbin 2009), retaining only alignments with a mapping quality of > 30. Variant calling was performed using GATK 3.7. We first called variants within individuals per chromosome using the HaplotypeCaller module with a custom slurm array script in order to parallelise jobs on our computing cluster. Per‐chromosome individual GVCFs were then merged into a single individual GVCF and jointly genotyped using the GATK GenotypeGVCFs module. Because of the large number of samples and the large size of some chromosomes, we split the genome into 10 Mb windows and called each of these in parallel to produce multiple raw, unfiltered VCFs.

Following variant calling, we randomly sampled 100,000 sites from across the genome using vcflib‐random‐sample in order to determine the empirical distribution of variant statistics from the unfiltered VCF. These included QD (quality by depth), FS (Fisher strand bias), and MQ (mapping quality). By examining the distributions of these statistics, we determined thresholds suitable for our data, as recommended by the GATK best practice guidelines. We then ran the GATK VariantFiltration module to annotate our VCF with a hard‐pass filter ensuring that only sites with QD < 5.0, FS > 10.0, MQ < 40.0, MQRankSum < −2.5, and ReadPosRankSum < −2.5 were marked as passing.

To efficiently filter variants, we continued the parallelisation approach outlined above; that is, working on VCFs for 10 Mb genome windows. We first filtered all VCFs to remove spanning deletions and then normalised them using bcftools *v*1.9 (Danecek et al. 2021). We then used vcftools v0.1.16 (Danecek et al. 2011) and bcftools to further filter our data. Specifically our filters were a max missingness = 20%, minimum site‐quality Phred score = 30, minimum mean site depth = 5, maximum mean site depth = 30, minimum genotype depth = 5 and maximum genotype depth = 30.

### 2.7 Phylogenetic Analyses

We performed a Bayesian phylogenetic analysis based on whole‐genome data for seven species of the genus *Passer* ( *P. diffusus* , *P. melanurus* , *P. iagoensis* , *P. luteus* , *P. montanus* , *P. domesticus* , and *P. hispaniolensis* ) collected for this study alongside 10 other Passerine species, all of which were downloaded from NCBI or ENSEMBL: *Prunella himalayana* (Prunellidae; GCA_013398875.1), *Ploceus nigricollis* (Ploceidae; GCA_013399945.1), *Taeniopygia guttata* (Estrildidae; Taeniopygia_guttata.bTaeGut1_v1), *Motacilla alba* (Motacillidae; GCF_015832195.1), *Serinus canaria* (Fringillidae; Serinus_canaria.SCA1), *Camarhynchus parvulus* (Thraupidae; GCA_902806625.1), *Emberiza fucata* (Emberizidae; GCA_013398835.1). We also included the following members of Passeridae to break the longer branch leading to the crown *Passer*: *Hypocryptadius cinnamomeus* (GCA_013400635.1), *Montifringilla adamsi* (GCA_017590115.1), and *Pyrgilauda ruficollis* (GCF_017590135.1).

After masking repetitive regions in all 17 genome assemblies with RED v2015‐05‐22 (Girgis 2015), we produced a whole‐genome alignment with CACTUS v.1.3.1 (Armstrong et al. 2020). We then extracted 10,000 alignment blocks of a length of 1000 bp from this whole‐genome alignment, with a minimum completeness of 95%, a minimum number of 10 polymorphic sites, and with only one sequence per species in each block. To identify the phylogenetically most suitable alignment blocks, we inferred phylogenies for each individual block under maximum likelihood, using the program IQ‐TREE v.2.1.2 (Minh et al. 2020) and applying the general time‐reversible (GTR) model (Tavaré 1976) with four gamma‐distributed rate categories. Separately, we concatenated all alignments and inferred the phylogeny from the concatenated alignment, again using IQ‐TREE v.2.1.2 but now applying the GTR model with 10 rate categories. Per‐block phylogenies were then compared to the phylogeny based on the concatenated alignment by measuring their Robinson‐Foulds (RF) (Robinson and Foulds 1981) distances with the help of the ETE3 python library (Huerta‐Cepas et al. 2016). We further characterised alignment blocks by calculating their numbers of variable sites and their hemiplasy score. To calculate the latter, we compared the numbers of variable sites and the parsimony score, determined with PAUP* v.4.0a163 (Swofford 2004) as done in (Ronco et al. 2021). We proceeded by selecting all alignment blocks with between 100 and 250 variable sites and less than 50 hemiplasies for further analysis (a total of 2005 alignment blocks). For each of these blocks, we used BEAST v.2.6.4 (Bouckaert et al. 2019) to apply Bayesian phylogenetic inference under a relaxed‐clock model (Drummond et al. 2006), combined with the Hasegawa‐Kishino‐Yano (HKY) (Hasegawa et al. 1985) substitution model and a (Udny Yule 1925) tree prior. For each block, we performed Markov‐chain Monte Carlo (MCMC) with a length of 5,000,000 iterations. Following this analysis, we quantified the parameters minimum effective sample size (ESS), mean Bayesian posterior probability (BPP) node support, and mean and standard deviation of the mutation rate estimates. We identified the most suitable alignments for a species‐tree analysis as those that had a minimum ESS value greater than 200, a mean BPP greater than 0.8, as well as intermediate mean mutation rates (between the first and third quartile), and a low standard deviation of the mutation rate (within the first quartile); these thresholds were met by a total of 153 alignment blocks. These 153 alignment blocks were then used jointly to infer the species tree under the multi‐species coalescent model with the StarBEAST2 version 1.0.0 method (Ogilvie et al. 2017), available through BEAST2. In this inference, we assumed a constant population size across branches and time, independent HKY substitution models for each alignment block, and the birth‐death tree prior as a model of diversification (Gernhard 2008). The species tree was time‐calibrated according to the timeline of bird diversification inferred by (Claramunt and Cracraft 2015), by constraining the root of the phylogeny (the age of the first divergence within Passeroidea) with a lognormal distribution with a mean of 28.8236 Ma and a standard deviation of 0.1. We performed five replicate StarBEAST2 analyses, each with a MCMC length of 1.5 billion iterations. We determined the burn‐in proportion of each MCMC by visualising traces in the program Tracer 1.7 (Rambaut et al. 2018), and then combined the post‐burnin proportions of three chains that could be considered stationary. Finally, a maximum‐clade‐credibility summary tree was produced from the combined MCMC chains with TreeAnnotator (part of the BEAST2 package; Heled and Bouckaert 2013). The age inferred in this tree for the most recent common ancestor of *P. montanus* , *P. domesticus* , and *P. iagoensis* (mean: 5.3286 Ma; 95% highest posterior density interval: 5.9293–4.6762) was then used to define an age constraint in the second phylogenetic inference, in which these three species were jointly analysed based on SNP data.

For the estimates of population divergence in Iago Sparrow, we began by subsetting variant call files, selecting a maximum of five randomly selected samples per population or species. We then applied Bayesian phylogenetic inference with SNAPP 1.6.1 (Bryant et al. 2012), implemented in the program BEAST2 v2.7.3. Following Stange et al. (2018), we used the script snapp_prep.rb (https://github.com/mmatschiner/snapp_prep) to prepare XML input files for SNAPP, and thus implemented a molecular‐clock model for the inference of time‐calibrated trees. In a first SNP‐based analysis, we aimed to infer the time of the onset of diversification among the more divergent populations of Iago sparrows, by using a dataset that also included samples of House and Eurasian Tree sparrows, and by constraining the divergence time of these two species according to results of the StarBEAST2 analysis described above. This constraint was implemented through a lognormal prior distribution with a mean of 5.3286 Ma and a standard deviation of 0.06. To achieve feasible run times of the computationally demanding SNAPP analyses, the dataset used in this analysis consisted of 1000 randomly selected bi‐allelic SNPs with a minimum chromosomal distance of 100 bp. The inferred age of the earliest diversification among sampled populations of the Iago sparrow was then used to time‐calibrate a second SNP‐based phylogeny based on RADseq data. This dataset included more populations of the Iago sparrow, but excluded the outgroup species (house and Eurasian tree sparrows). The age constraint was again implemented through a log‐normal prior distribution on the age of the root of the phylogeny, this time with a mean of 0.884 Ma and a standard deviation of 0.1. The input datasets were, as in the first SNP‐based analysis, reduced to a maximum of five randomly selected samples per population and 1000 randomly selected bi‐allelic SNPs, separated from each other by a minimum distance of 100 bp. Both analyses used five replicate Markov‐Chain Monte Carlo (MCMC) chains with an initial length of 100,000 iterations. Upon completion, these chains were assessed for convergence and stationarity by inspecting parameter traces and effective sample size (ESS) values using Tracer. The chains were resumed until all ESS values of individual chains were above 100, in which case replicate chains were merged into a single posterior distribution per analysis. Summary trees were generated as for the StarBEAST2 1.0.0 analysis with TreeAnnotator.

### 2.8 Population Genomic Analyses

For population structure analyses we started by identifying sites called only in the RADseq dataset and then we extracted them from the whole genome data. The two datasets were subsequently merged using bcftools and the merged data was further pruned for linkage disequilibrium using PLINK 1.96 (Purcell et al. 2007), specifying trimming data with a linkage above 0.10. We then used PLINK to do the PCA and ADMIXTURE v1.3.0 (Alexander et al. 2009), and plotted these with ggplot on R (Irizarry 2024).

We used the RADseq data only for estimating spatial variation in effective dispersal rate with the Estimating Effective Migration Surfaces (EEMS) software (Petkova et al. 2016). EEMS identify changes in effective dispersal rate at a large geographical scale due to, for instance, the presence of a physical barrier or to unsuitable environmental conditions for dispersal. EEMS is based on a two‐dimensional stepping‐stone model, estimates effective migration rates between adjacent subpopulations (demes) and does not require prior knowledge on the landscape features that could influence gene flow. Following Petkova et al. (2016), we used the genetic dissimilarity matrix generated by the EEMS program *bed2diffs_v2* and raw geographic coordinates for each sampling site as input files for EEMS. We ran EEMS with different grids, differing in number of demes, to investigate how and if results would change with grid size. We tested several numbers of demes, namely 200, 400, 600 and 800 demes, and checked whether results would change across deme size. We performed the analysis eight times (eight chains) using MCMC length of 8 × 10^6^ iterations with a burn‐in of 10^6^ iterations and set 999 as the number of iterations to thin between two writing steps. To ensure MCMC convergence and reliable parameters estimation, we checked for convergence in the posterior trace plot generated in rEEMSplot package and made sure that the parameters values of the models would be accepted about 20%–30% of the time, or less than 10% (Petkova et al. 2016). We then combined results from different chains using the rEEMSplot package and plotted surfaces of effective migration rates.

We phased biallelic variant sites using shapeit2.r904. Phasing was performed using default parameters on 10 Mb windows across the genome. We took the house sparrow recombination map as a means of informing the phasing algorithm (Hagen et al. 2020). Phased windows were then combined into per chromosome binary variant call formats using the shapeit5 ligate utility.

We calculated F_ST_, d_XY_ and genetic diversity (π) using the genomics general scripts by Simon Martin (https://github.com/simonhmartin/genomics_general), converting the data to ‘geno’ format and then running these analyses in windows of 10,000 bp with steps of 2500 bp. Our window size was selected based on the analysis of the genome‐wide level of linkage disequilibrium decay using PopLDdecay (Zhang et al. 2019). We used an outlier approach to identify regions of the genome putatively under selection; in short we identified windows with F_ST_ greater than the 97.5th percentile. After this, we used the phased data biallelic SNP data to calculate iHS (Integrated Haplotype Score) and Cross Population Extended Haplotype Homozogysity (XP‐EHH) for the regions on chromosome 28 and chromosome 1A using rehh (Gautier et al. 2017).

We used the ancestral recombination graph (ARG) inference method *tsinfer + tsdate* by estimating within‐group TMRCA (Kelleher et al. 2019; Wohns et al. 2022) to infer tree sequences and date tree nodes. Biallelic variants from the phased VCF files were converted to Zarr format with the vcf2zarr conversion utility (Czech et al. 2025). We assigned ancestral states to segregating sites by polarising with respect to tree sparrows as the outgroup by applying a maximum‐likelihood method implemented in FastDFE (Sendrowski 2024) and ran the tsinfer inference workflow. We dated nodes with tsdate, assuming a mutation rate of 2.3 ⨉ 10^−9^ mutations per site per year (Smeds et al. 2016) and a recombination rate of 1.5 ⨉ 10^−8^ (Backström et al. 2010). The resulting tree sequences were analysed with the *tskit* library https://paperpile.com/c/VAyh5G/NeMp (Kelleher et al. 2016; Wong et al. 2024). For each tree sequence, we extracted the node and its age of the most recent common ancestor (MRCA) for all of the focal populations east, west, and Rombos, and plotted the age distributions with the Python package seaborn (Waskom 2021).

Population demographic histories were inferred with *PHLASH* using parameters window_size = 100, niter = 1000, and overlap = 500 (Terhorst 2024). Chromosome 19 was used as test data to avoid overfitting, as suggested by the documentation. We made population history plots with custom Python scripts. As PHLASH uses coalescent scaling, we rescaled the output after model fitting using a mutation rate of 2.3 ⨉ 10^−9^ mutations per site per year, assuming a generation time of 1 year.

## 3 Results

### 3.1 Evolutionary History of the Iago Sparrow

To provide a clearer evolutionary context on the Iago sparrow system, we first inferred a whole‐genome phylogeny for multiple *Passer* species. We estimated the time of divergence of the family Passeridae, the family to which sparrows belong, to be 27.25 million years and the genus *Passer* to be 8.28 million years old (Figure 1B). Our estimate for *Passer* divergence is older than (Päckert et al. 2021), who, using 2 nuclear and 2 mitochondrial markers, estimated the origin of the genus at approximately 6 million years ago. Those authors also found that the Iago sparrow was the sister lineage to a clade including the Sudan golden sparrow ( *P. luteus* ), the desert sparrow ( *P. simplex* ), and the Dead Sea sparrow ( *P. moabiticus* ). Our whole‐genome phylogeny is consistent with this relationship and estimated a divergence time of 4.26 million years between the Iago sparrow and the Sudan golden sparrow (Figure 1B), consistent with the estimate of approximately 3.9 million years reported by (Päckert et al. 2021). Divergence between the house ( *P. domesticus* ) and the Spanish sparrows ( *P. hispaniolensis* ) was 1.43 million years (Figure 1B), similar to 1.29 million years uncovered by (Päckert et al. 2021), but older than other estimates based on demographic analyses incorporating gene flow (Ravinet et al. 2018).

Our whole‐genome phylogeny of the Passeridae provides a time frame for when the Iago sparrow may have become endemic on the Cabo Verde archipelago—that is, sometime within the last 4.3 million years. We also performed phylogenetic analyses across Iago sparrow populations to investigate temporal dynamics within the archipelago. To balance sample size and genome coverage we analysed RADseq and whole‐genome resequenced datasets. These analyses indicate that divergence between *P. iagoensis* populations occurred within the past 1 million years, with the separation of the Rombos islets lineage (for geographical location see map in Figure 2A) splitting from the remaining lineages around 0.87–0.88 million years ago (Figure 1C,D). The second major split in the Iago sparrow population tree, estimated to have occurred 0.5 million years ago (Figure 1C,D), separates birds from Raso, São Vicente, and Santa Luzia—historically a single landmass during glacial sea‐level lowstands (Ramalho 2011)—from those inhabiting the remaining islands (Brava, São Nicolau, Santo Antão, Sal, Boavista, Maio, and Santiago). There is topological discordance with the positioning of Brava between RADseq and whole‐genome datasets: in the RADseq phylogeny Brava is reconstructed as the sister lineage to all remaining lineages (Sal, Boavista, Santiago, Maio, São Nicolau and Santo Antão; Figure 1C), while the whole‐genome tree shows Brava as sister only to São Nicolau and Santo Antão (Figure 1D). Importantly, divergence times among *P. iagoensis* lineages estimated from RADseq data are consistent with those estimated from whole‐genome data.

**FIGURE 2:**
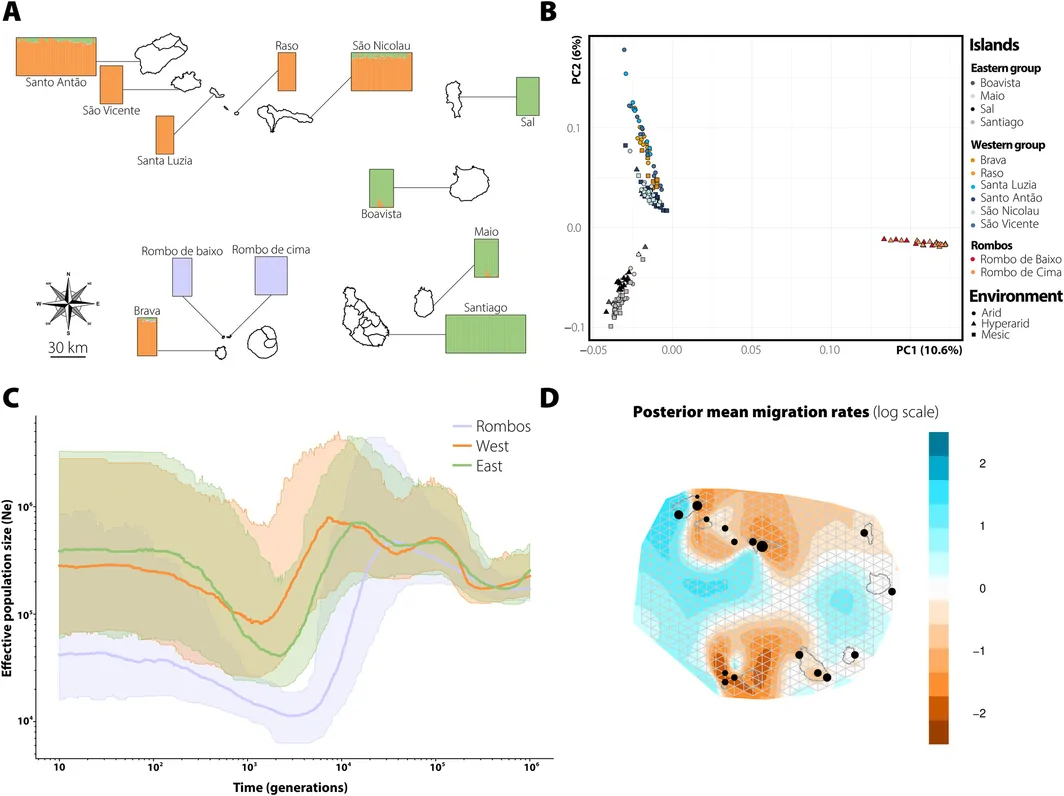
Population structure and demographic change in the Iago sparrow. (A) Admixture proportions plotted over a map of Cabo Verde (ddRADseq data). The most supported cluster, *K* = 3, is shown. (B) Principal Component Analysis of genomic data. Islands are colour‐coded, and the different environment types are shown by different symbols (arid, hyperarid and mesic as squares, triangles and circles, respectively; see methods for details). (C) Effective population size (Ne) through time (generations) estimated with PHLASH. Shading indicates confidence intervals, lines denote median estimates after 1000 iterations. (D) Estimated Effective Migration Surfaces (EEMS) showing relative migration rate across islands and sampling sites (black dots; nearest node of grid) using a 200 demes grid.

### 3.2 Population Structure in the Cabo Verde Archipelago

The Cabo Verde archipelago consists of 10 major environmentally diverse islands and 4 major islets. The Iago sparrow is present on 9 of the main islands as well as three of the major islets. Therefore, we investigated whether these spatial and environmental patterns are reflected in the population structure of this endemic species. We first analysed a large dataset encompassing 234 individuals (Table S1) derived from a combination of RADseq (224 individuals) and whole‐genome resequencing (43 individuals). A subset of 33 individuals underwent both approaches allowing us to control for batch effects (Table S1). Admixture (Figure 2A) and principal component analyses (PCA; Figure 2B) clustered all 234 individuals into three distinct population groups: the Western islands (Santo Antão, São Vicente, Santa Luzia, Raso, São Nicolau, and Brava), the Eastern islands (Sal, Boavista, Maio, and Santiago), and the Rombos islets (Ilhéu Grande and Ilhéu de Cima; situated near Fogo and Brava). The PCA separated birds from Rombos versus the remaining populations along the first component (PC1), which accounted for 10.6% of the variation. PC2 (6% of variation explained) distinguished birds from the Western and Eastern islands. This partitioning of the data into three clusters is consistent with an optimal value of *K* = 3 in ADMIXTURE analyses (Figures S1 and 2A). We note that this contrasts with the only previous mtDNA study which found no evidence for geographical structuring in the Iago sparrow (Päckert et al. 2021).

Estimates of effective population size (Ne) using PHLASH indicated that the three lineages have likely undergone a bottleneck around 7,000–11,000 generations ago. The strongest and oldest of these bottlenecks was observed in the Rombos populations (Figure 2C). PHLASH analysis also suggests that the separation of the lineages occurred on the order of several hundred thousand years ago (Figure 2C) and shows that after a more recent bottleneck Rombos has maintained an Ne that is at least an order of magnitude smaller than the other main lineages. To investigate how geography influences genetic variation and connectivity, we estimated effective migration surfaces (EEMS). EEMS revealed that gene flow patterns are more strongly linked to the geographical distance between islands, with the striking exception of the Rombos population which is strongly differentiated despite being only ~7 km from the nearest population at Brava (Figure 2D).

### 3.3 Phenotypic Evidence of Divergent Selection in Bill Morphology

The Iago sparrow is widespread across Cabo Verde's environmentally heterogeneous archipelago, which ranges from arid to mesic habitats. Given the strong structure among island groups revealed by our phylogenetic and population genomic analyses, we next asked whether the genetic and environmental heterogeneity explains phenotypic variation across islands and the broader aridity gradient within the archipelago. To achieve this, we collected five linear morphological measurements from 374 individuals, including 165 females and 209 males (Table S2). Using general linear models, we found sexual dimorphism for all six measured traits, with smaller mean values in females (Table 1A, factor: sex). These models also revealed significant differences in all traits between birds from different islands (Table 1A). However, there was no significant sex*island interaction, indicating that females were smaller than males regardless of geographical location (Figure S2). We therefore accounted for sexual dimorphism in further analyses.

**TABLE 1:** Morphological divergence and sexual dimorphism.

			Wing length	Tarsus length	Bill length	Bill depth	Bill width	Bill volume
A	Sex		F_(1,346)_ = 739.42***	F_(1,346)_ = 357.95***	F_(1,346)_ = 786.72***	F_(1,346)_ = 1285.7***	F_(1,346)_ = 632.71***	F_(1,346)_ = 1682.10***
	Island		F_(11,346)_ = 13.93***	F_(11,346)_ = 27.35**	F_(11,346)_ = 27.32**	F_(11,346)_ = 46.03**	F_(11,346)_ = 14.66***	F_(11,346)_ = 48.40***
	Island × sex		F_(11,346)_ = 0.50	F_(11,346)_ = 1.19	F_(11,346)_ = 1.18	F_(11,346)_ = 1.64	F_(11,346)_ = 1.31	F_(11,346)_ = 1.47
B	Genetic cluster	♀	East‐Rombos***	East–Rombos***	East–Rombos**	East–Rombos***	East–Rombos***	East–Rombos***
			East–West ns	East–West**	East–West***	East–West***	East–West ns	East–West***
			Rombos–West***	Rombos–West***	Rombos–West***	Rombos–West***	Rombos–West***	Rombos–West***
		♂	East–Rombos***	East–Rombos***	East–Rombos**	East–Rombos***	East–Rombos***	East–Rombos***
			East–West ns	East–West ns	East–West***	East–West***	East–West***	East–West***
			Rombos–West***	Rombos–West***	Rombos–West***	Rombos–West***	Rombos–West***	Rombos–West***
C	Climate PC1		F_(1,366)_ = 10.47**	F_(1,366)_ = 18.87***	F_(1,366)_ = 14.12***	F_(1,366)_ = 39.26***	F_(1,366)_ = 19.71***	F_(1,366)_ = 33.81***
	Climate PC2		F_(1,366)_ = 278.02***	F_(1,366)_ = 8.34**	F_(1,366)_ = 0.24 ns	F_(1,366)_ = 0.32 ns	F_(1,366)_ = 8.19**	F_(1,366)_ = 2.27 ns

*Note:* (A) Effect of island (*n* = 12) and sex (F; M) on morphological variation as revealed by a two‐way ANOVA. (B) Effect of genetic population cluster (*n* = 3): post hoc Tukey's HSD test after significant ANOVA (see Figure 3). (C) ANOVA results after MANOVA (morphology ~ PC1 + PC2 + sex) revealed a significant effect of the two climatic axes on morphological traits.

** *p* < 0.01.

*** *p* < 0.001.

We analysed phenotypic variation among the three population clusters identified in the population structure analysis: Rombos, West and East (Figure 2A). All six traits displayed significant differences, indicating clear morphological distinctions among the lineages (F_(2,363)_ = 1.58, *p*‐value < 0.001). Post hoc tests revealed that birds (both males and females) from the East and West clusters are challenging to differentiate based on wing length. For tarsus length and bill width, significant differences were observed in one sex only—females (tarsus length) and males (bill width), respectively (Table 1B, Figure 3A). All comparisons involving birds from the Rombos islets were significant (Table 1B), with a general trend toward larger birds in the Rombos cluster (Figure 3A).

**FIGURE 3:**
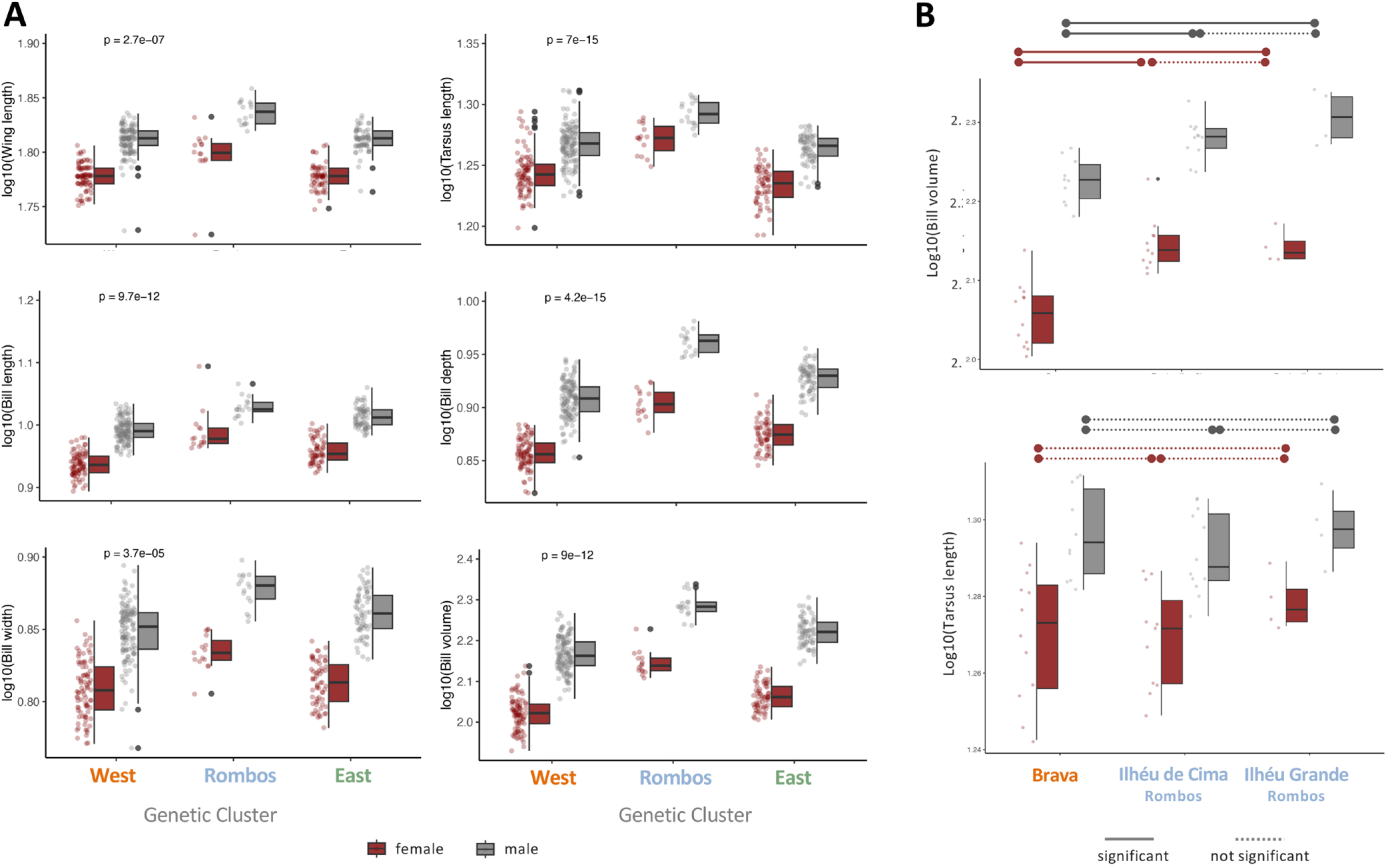
Morphological divergence among Iago sparrow populations across the Cabo Verde archipelago. (A) Differences among genetic population clusters: West, Rombos, East; *p*‐values reported for ANOVA: Morphology ~ population cluster + sex; pairwise comparisons in Table 1B. (B) Divergence in bill volume and tarsus‐length between sparrows from Brava and Rombos islets, located ~7 km apart. Solid lines indicate statistically significant differences and dashed lines depict non‐significant comparisons (Holm‐adjusted *p*‐values after *t*‐test).

To assess the role of environmental heterogeneity in shaping phenotypic variation, we investigated whether there was any association between climatic variables and phenotypic traits. To this end, we characterised the climatic context of the sampled populations (see Figure S3). There was a significant effect of PC1 and PC2 (MANOVA; Wilk's = 0.904, *p* = 0.001, Wilk's = 0.938, *p* < 0.001, respectively). Individual linear models on these two climate variables suggest that all traits correlate with PC1 (reflecting differences in precipitation regimes: annual precipitation (Bio12) and precipitation of the wettest quarter (Bio16)), while only wing and tarsus length correlate with PC2 (reflects the effect of temperature seasonality: mean temperature of the coldest quarter (Bio11) and mean annual temperature (Bio1)) (Table 1C).

Finally, we focused on birds from the Rombos islets. These birds are the most genetically and morphologically distinct within the archipelago and thus likely have experienced divergent selection compared to other populations. Both sexes from Rombos exhibited significantly larger bill volumes compared to all other populations (mean values by sex across the species' entire range; Figure S2). Considering the potential role of the bill in thermoregulation and foraging (Ryeland et al. 2017), we compared bill volumes of birds from Rombos to those from Brava, which, despite its geographic proximity (~7 km), differs environmentally (hyper‐arid vs. mesic, respectively). This analysis revealed no within‐group differences in bill volume among Rombos birds but showed a significant increase in bill size compared to Brava for both sexes (Figure 3B, top panel).

### 3.4 Genomic Signatures of Selection Associated With Divergent Bill Phenotypes

Given the phenotypic divergence in bill size between Iago sparrows from the Rombos islets and elsewhere in Cabo Verde, we used our whole‐genome resequencing dataset to investigate genetic signatures of divergent natural selection. First, we used whole‐genome resequencing data to test for divergent selection across traits, including bill volume, via P_ST_ vs. F_ST_ analysis (Figure S4). Finding clear evidence for divergence, we next proceeded to identify the genomic basis of this divergent phenotype. We obtained pairwise sliding‐window estimates of genomic differentiation (F_ST_), divergence (D_XY_), long‐range haplotype statistics (*XP‐EHH*; *iHS*), and diversity (π). We scanned for outlier regions (top 2.5% F_ST_), focusing on Rombos vs. West and Rombos vs East. We found two genes of major relevance to bill morphology among all genes in the top 2.5% F_ST_ outlier regions and that may play a role in underlying the large bill volumes seen on the Rombos islets (Figure 3). The first was *ALX1* on chromosome 1A, which has been implicated with bill shape variation in Darwin's finches (Lamichhaney et al. 2015) and great tits (Bosse et al. 2017). The second was *HMG20B*, High mobility group 20B, a gene related to *HMG4* on chromosome 28 and which is associated with changes in bill size (Lamichhaney et al. 2015). Pairwise divergence estimates (D_XY_) between West and Rombos for the regions associated with *ALX1* and *HMG20B* was not higher than the background genomic, but diversity (π) in Rombos decreases in these regions (Figure 4A). Both *ALX1* and *HMG20B* show evidence of increased extended haplotype homozygosity (i.e., xpEHH, Figure 4C), though the evidence for *HMG20B* is clearer. Consistent with this, *ALX1* and *HMG20B* haplotype length was significantly larger in Rombos when compared to the West populations (Figure S5). The same pattern (i.e., larger haplotypes in Rombos) hold when compared to two random regions in the same chromosome for *ALX1* and one random region in the same chromosome for *HMG20B* (Figure S5). Finally, we performed a P_ST_ vs. F_ST_ analysis to formally test for divergent selection, this time focusing on our outlier genes. We found that divergent selection is indeed likely occurring (P_ST_ > F_ST_) and is furthermore likely sufficient to counteract gene flow among island groups (Figure S4).

**FIGURE 4:**
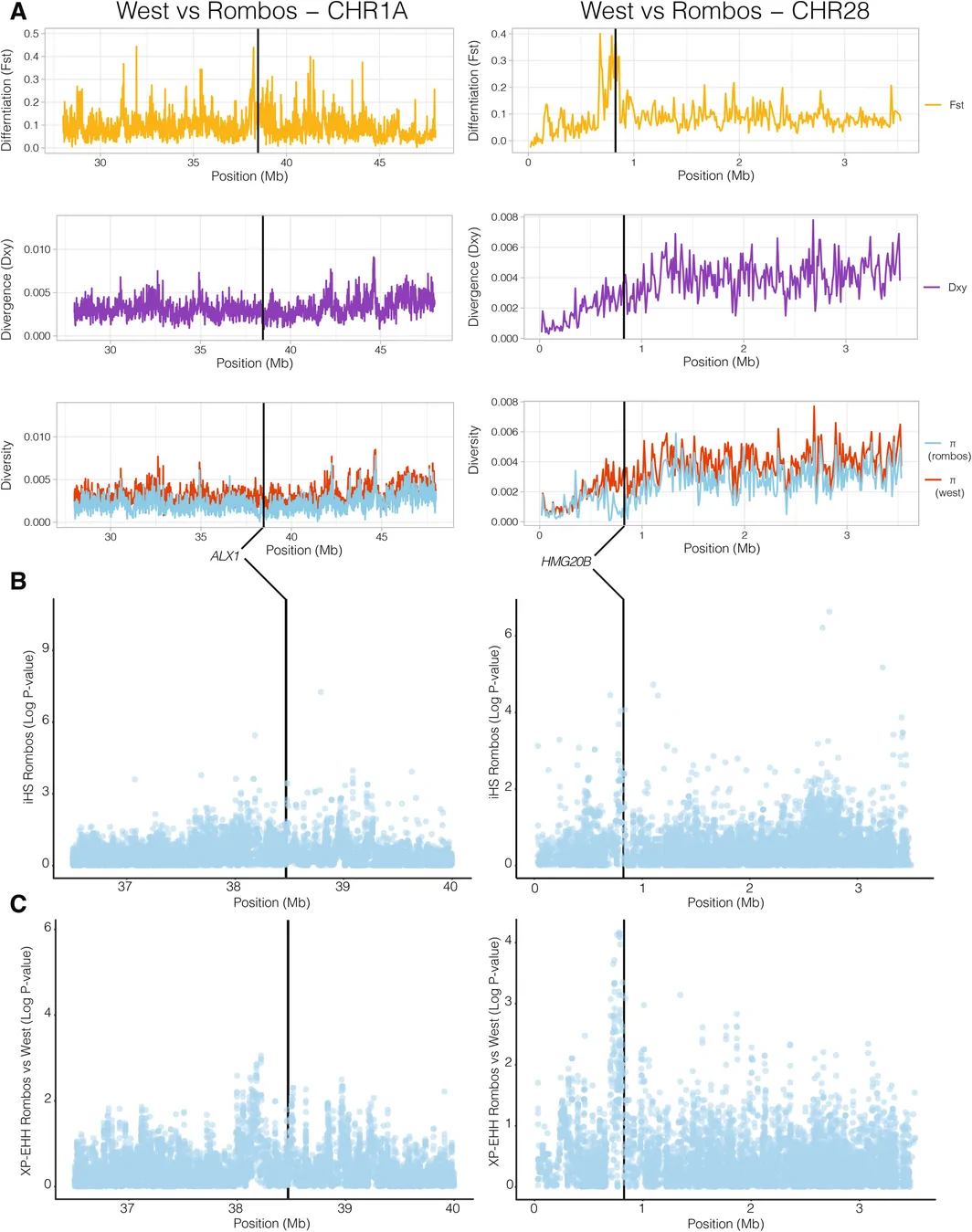
Exploration of genomic signatures of selection for divergence in bill morphology between Iago sparrow populations at two putative large‐effect genes involved in beak size and shape, *ALX1* (left) and *HMG20B* (right). (A) Genomic differentiation (F_ST_), genomic divergence (D_XY_) and genetic diversity in windows (π). F_ST_ and d_XY_ for the comparison Rombos vs West. The vertical lines mark the location of *ALX1* in chromosome 1A and *HMG20B* in chromosome 28. (B) Log transformed *p*‐value of iHS (Integrated Haplotype Score). Chromosome 1A:37–40 Mb region; black line indicates *ALX1*. Chromosome 28: Full chromosome; black line corresponds to *HMG20B*. (C) Log transformed *p*‐value of Cross Population Extended Haplotype Homozygosity (XP‐EHH). Same genomic regions and gene markers as in (B).

### 3.5 Age of Adaptive Allelic Variation

Our ARG analysis estimated colonisation of the Cabo Verde archipelago by the Iago sparrow, confirmed genetic structuring among island groups, and detected evidence of phenotypic and genomic selection on functionally relevant traits. We used an ARG approach to estimate the age of allelic variation across the genome to determine whether variation linked to adaptation arose prior to or following colonisation. This has the advantage of allowing us to efficiently estimate genome‐wide genealogies among a sample of individuals and then estimate a TMRCA on these genealogies—effectively providing an estimate of when variation first arose (Lewanski et al. 2024; Wohns et al. 2022). To do this, we used *tsinfer* to infer the ancestral recombination graph followed by *tsdate* to estimate the divergence times of haplotypes on chromosomes 1A and 28. The distribution of genealogical ages showed a similar pattern for chromosomes 1A and 28 (Figure 5). All populations exhibited two peaks in haplotype age. For chromosome 1A, the rightmost peak (oldest) was consistently positioned at 2.07 million years ago (mya) across all populations (Figure 5A), while the younger peaks (left) occurred at 0.53, 0.51, and 0.49 mya in the West, East, and Rombos lineages, respectively. Similarly, for chromosome 28, the oldest peak was found at 2.05 mya in all populations (Figure 5B), with the younger peaks at 0.39 mya in the Western group, 0.4 mya in the Eastern group, and 0.36 mya in the Rombos population. The estimated ages for both the *ALX1* and *HMG20B* haplotypes in Rombos are much more recent than in the other populations, concordant with a scenario of recent evolution followed by selection.

**FIGURE 5:**
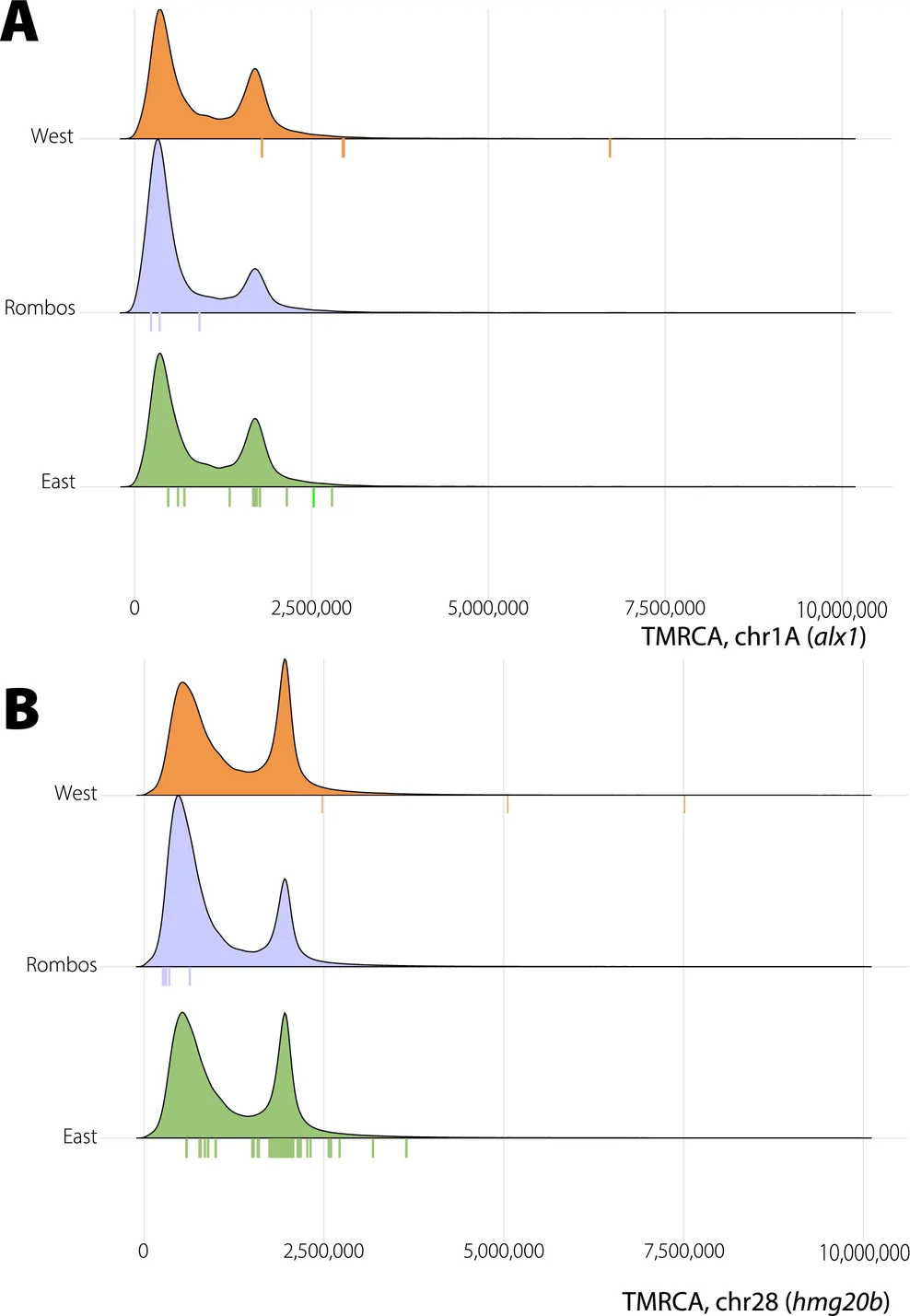
Distributions of the age of allelic variation at candidate regions underlying bill morphology differences in the Iago sparrow. Time to the most recent common ancestor (TMRCA) for (A) Chromosome 1A, *ALX1*; (B) Chromosome 28, *HMG20B*. The age of each haplotype is given for the three lineages, as frequency distributions. Vertical lines under the X axes highlight the estimated ages of the haplotypes in the genes *ALX1* (A) and *HMG20B* (B) for each lineage.

## 4 Discussion

How important is ancestral variation in adaptation? Expanding the range of studies across insular lineages offers valuable insights into how this ancestral variation can shape the evolution and maintenance of genetic diversity in colonising populations. We studied the Iago sparrow, a species endemic to the Cabo Verde archipelago, whose distribution spans a wide range of environments, including those altered by human activity to investigate the potential role of ancestral variation in facilitating its ecological breadth. Our findings show population structure, with three main lineages diverging approximately 0.87–0.88 million years ago (Figures 1C,D and 2A,B). Colonisation of the archipelago likely occurred sometime between ~4.26 and 0.88 million years ago, with the upper limit derived from phylogenomic divergence from its closest relative *Passer luteus* (Figure 1B), and ARG analysis suggest that colonization occurred around 2 million years ago. We also found strong phenotypic and genomic evidence of divergent selection driving adaptive variation among Iago sparrow lineages. Sparrows from the Rombos islets exhibited significantly larger bill and body sizes (Figure 3) with genomic signatures of selection at two genes associated with bill size and shape (*HMG20B* & ALX1; Figure 4). Our genome‐wide genealogical analysis indicated that *HMG20B* haplotypes occurring in Rombos had a recent TMRCA (Figure 5), suggesting that variation underlying adaptive divergence arose within the archipelago but is now restricted to the lineage with the smallest population size and geographical range, and occupying rather harsh environmental conditions. These results provide important insights for the evolutionary dynamics of island systems: novel adaptive variation in beak traits likely evolved within the archipelago and persisted despite gene flow and bottlenecks.

### 4.1 The Geography of Cabo Verde Has Shaped Sparrow Evolution

One of our most striking findings is the deep divergence (0.87–0.88 Mya) among the three lineages of Iago sparrow within the Cabo Verde archipelago, contrasting with a previous study based on mitochondrial markers that found no phylogeographic structure (Päckert et al. 2021). We had initially hypothesised that climate was a key driver of population genetic structure. Although it likely does play some role, geography is apparently a more important determinant: the two larger clades of the Iago sparrow, eastern and western, mirror a clear geographic divide. In contrast to our original expectations, both the eastern and western clades occupy areas with highly variable precipitation regimes and temperature ranges. For instance, in Brava, Santo Antão and São Nicolau (western), and in Santiago (eastern), we sampled birds living in both arid and humid subtropical areas. Further evidence for geography as a driver of structure is also provided by high migration rates between neighbouring islands (EEMS; Figure 2).

The evolutionary history of the Iago sparrow postdates the geological age of the archipelago (formed between 15 and 25 million years ago; Madeira et al. 2008; Cunha et al. 2017). However, our phylogenomic analyses date its divergence from its sister lineage ( *P. luteus* ; but see Päckert et al. 2021) to approximately 4.26 million years ago. Furthermore, a single colonisation event in the early Pleistocene, around 2 million years ago, is suggested by the ARG analysis (Figure 5), which shows a peak in TMRCA for haplotypes across all populations at this time point and chromosomes (Figure S6). Such a peak typically indicates that many haplotypes originated from a specific period, potentially due to an expansion following a founder effect. However, further work is necessary to distinguish this from the mean time of coalescence of haplotypes within the Iago population. Furthermore, we cannot exclude the possibility that the Iago sparrow is the relict of an extinct widespread lineage (paleoendemic) from western Africa, that may have diverged from its sister clade (encompassing the Sudan golden, desert and Dead Sea sparrows). In any case, the large time gap between island age and colonisation, and the divergence within extant Iago sparrow populations is similar to observations in other oceanic islands. For example, Príncipe Island is approximately 30 million years old but divergence times for nearly all endemic bird species date back to around 2.5 million years, with the oldest known species dating back to 8 million years (Melo et al. 2022). Notably, in the Iago system, inconsistencies between phylogenetic analyses suggest possible intra‐archipelago migration events within the last 0.51 million years, coinciding with the divergence of all east–west lineages (Figure 2). This interpretation is further supported by our admixture analyses, which show minor ancestry components shared between eastern and western populations. A decline in Ne occurred approximately 10,000 years ago, coinciding with post‐glacial sea level rise, when several large islands fragmented into smaller ones (Lambeck and Chappell 2001). However, we remain cautious with this interpretation as population sizes inferred with approaches such as PHLASH may be overestimated by ancient genetic diversity and additional fine‐scale population structure (Steux et al. 2025).

### 4.2 Selection Shapes Adaptive Divergence Within the Iago Sparrow

Our genomic analyses identified a highly divergent Iago sparrow lineage on the Rombos islets (approximately 1 km^2^; desert‐like environment with high aridity, sparse vegetation and limited surface water sources) in the south west of the archipelago. Given this environmental setting, we expected that natural selection is likely to be driving phenotypic divergence between birds from the Rombos and elsewhere in the archipelago.

While accounting for sexual dimorphism, Rombos birds have the largest body sizes and substantially increased bill volumes. Our genome scans identified two candidate genes (Figure 4): *ALX1*, implicated in bill shape changes, and *HMG20B*, a gene related to a gene linked to bill size variation in the Darwin's finches (Lamichhaney et al. 2015; Han et al. 2017; Rubin et al. 2022) (Figures S7 and S8). While extended haplotype statistics and coalescence times (Figure 5) showed signatures of selection for both genes, although the signal is strongest for *HMG20B*, P_ST_‐F_ST_ analyses provide support for the hypothesis that divergent selection on bill traits is strong enough to counteract the effects of gene flow and drift (Figure S5).

Bill variation is known to occur within the *Passer* genus (Eroukhmanoff et al. 2013, 2014; Runemark et al. 2018) and is associated with dietary divergence (Fawthrop et al., in preparation). Given the extreme environment of the Rombos, bill divergence likely reflects either adaptation to novel food resources or thermoregulatory selection due to higher temperatures, less shade, and high aridity (Friedman et al. 2017, 2019). At present, we are unable to distinguish between these two potential selective pressures, and further research on diet using direct observations and stable isotope analysis, as well as thermal physiology experiments, are needed to test these hypotheses.

### 4.3 The Evolutionary Dynamics of Insular Populations

Understanding the dynamics of island colonisation and adaptation is a fundamental question in island biogeography. Insights from a wide variety of species, especially from environmental generalists, are necessary to address this and the Cabo Verde sparrow system provides a valuable new case study.

We identified three divergent lineages within *Passer iagoensis* broadly consistent with the geographical setting of the archipelago but, notably, a 0.4 million years lag separates the initial split of Rombos lineage from further divergence, with evidence of more recent and rapid divergence of populations within the East and West lineages (Figure 1). This is surprising considering the contemporary archipelago‐wide geographic distribution of the species and the lack of obvious barriers to dispersal. For example, house sparrows are capable of dispersing long distances over water in archipelago systems (Ranke et al. 2024). It is therefore reasonable to assume that Iago sparrows have similar abilities given the observation of individuals undertaking extremely long‐distance journeys on cruise ships (Ebels et al. 2014).

Why, then, has divergence only occurred recently? One potential explanation for this is that ephemeral population dynamics, characterised by repeated extinctions and recolonisations of islands, may have played a significant role in shaping diversity with the Iago sparrow (Pereira and Singhal 2022; Rosenblum et al. 2012). Volcanic activity and extreme climate oscillations are a potentially important driver of local extinctions, preventing deep branching in our population tree, a pattern supported by the absence of Iago sparrows on Fogo, the volcanically active island. Therefore, it is quite likely that population diversification has occurred throughout the evolutionary history of the Iago sparrow on Cabo Verde but only surviving lineages are detectable nowadays. Extinction and ephemerality might also explain the incongruence of Brava clustering with geographically distant islands and inconsistent placement in different phylogenetic analyses (Figure 1C,D). It is possible that the western group was historically more widespread but later contracted, leaving Brava as a distant, isolated population. This can also explain Rombos' unexpectedly large effective population size, despite the islets' tiny area (300 and 150 ha, respectively). Specifically, the Rombos lineage may have once been more widespread but is now restricted in its range. More broadly, ephemeral populations may be a pervasive phenomenon in oceanic islands, as seen in the Gulf of Guinea island, where volcanic activity and extinction shaped avifauna diversity (Melo et al. 2022). Future research should focus on the role that ephemeral divergence and within‐archipelago metapopulation dynamics might play in maintaining variation for species colonising islands.

Related to these dynamics are outstanding questions on the age of genetic variation seen in island populations: is adaptation to new environments within archipelagos driven by standing genetic variation, that is, variation segregating in populations prior to colonisation? Or does this variation arise *de novo*, following colonisation of new environments? Our analyses on the Iago sparrow suggest that adaptation is likely driven by both processes simultaneously. We estimated a probable colonisation between 2.5 and 4.5 million years ago and the TMRCA of haplotypes within the Rombos is 2.5 million years. In contrast, our estimates of the age of the *HGM20B* region are much more recent at around 0.5 million years (Figure 5B). This indicates that the evolution of beak size changes in the Rombos population, likely associated with this gene, occurred after the species colonised the archipelago. In contrast, the region containing *ALX1*, associated with beak shape, presents a different pattern. Most *ALX1* genealogies fall within the overall distribution of genealogical ages, clustering around the two main TMRCA peaks for genealogies, although some regions show much older coalescence times (Figure 5A). Additionally, the distribution of genomic regions linked to *ALX1* closely matches the frequency distribution of the rest of the chromosome. To resolve these dynamics across island systems, incremental insights from diverse study systems beyond iconic radiations are crucial.

On a related historical note, it is fascinating that Darwin began his journey collecting sparrows in Cabo Verde (Santiago) and went on to collect finches in the Galápagos, only for us to discover that similar genes (*ALX1* and *HGM20B*) are under selection in both. This underscores the critical role of these genes in shaping the development and evolution of a key avian trait (and perhaps Charles Darwin's favourite trait): the bill.

## 5 Conclusion

We highlight the importance of integrating colonisation history and biogeography to further understand the dynamics of genetic variation and adaptation in island populations. We identified adaptive variants in the Iago sparrow associated with well‐known candidate genes related to beak morphology, consistent with phenotypic differences observed among populations. Our results indicate that the underlying genomic variation linked to bill divergence has likely arisen de novo within the archipelago and been shaped by standing genetic variation that may have pre‐dated colonisation. These findings contrast with recent studies in which ancient polymorphisms have driven adaptive radiation, such as in finches (Rubin et al. 2022), cichlid fishes (Meier et al. 2017), and *Metrosideros* plants (Choi et al. 2021). Further work is now necessary to better develop our understanding of this exciting island system, particularly adaptation to multiple selective pressures, including human‐modified environments and extreme aridity.

## Author Contributions

Â.M.R., M. Melo, P.C., A.F., E.P.L. collected data and obtained research permits. R.A.J., R.C.K.B., H.P., G.‐P.S., Z.O., and M.R. generated genetic data. J.C., Â.M.R., A.F., M. Melo, M.R. designed the research. P.E., T.A. contributed important samples. J.C., Â.M.R., M. Matschiner, G.D., P.U., Y.W., G.M.S., M.R. analysed the data. J.C. and Â.M.R. wrote the first version of the manuscript. All authors read and approved the submission.

## Funding

This work was supported by Fundação para a Ciência e Tecnologia (Grants EXPL/BIA‐EVF/2372/2013; UIDB/05937/2020, UIDP/05937/2020); EU MSCA Action REF: EVOSPARROW (Grant 748299); Peder Sather grant (‘Adaptation in the Anthropocene: the Iago Sparrow genome as a key to understand adaptation to anthropogenic environments’); SciLifeLab & Wallenberg Data Driven Life Science Program, Knut and Alice Wallenberg Foundation (Grants KAW 2020.0239 and KAW 2017.0003); Swedish Research Council through grant agreement no. 2022–06725. Fundaçao para a Ciencia e a Tecnologia, Portugal (Grants UIDB/05937/2020, UIDP/05937/2020).

## Ethics Statement

A permit for capturing, handling, and sampling sparrows, as well as to export biological material was issued by Direcção Geral do Ambiente (nb: 26/2014). Exportation to and importation of material into the USA (University of California, Berkeley) was authorised by the Instituto de Conservação da Natureza e das Florestas—ICNF (permit DE‐0040/2014) and US Department of Agriculture—APHIS (permit 115487‐Research), respectively.

## Conflicts of Interest

The authors declare no conflicts of interest.

## Supporting information


**Figure S1:** Our Special *K* On the Y axis we plot the error (Evanno‐method based) of different clusters (K). The most supported *K* = 2.
**Figure S2:** Sexual dimorphism in the six morphological traits across the entire range of the Iago sparrow. Females (red) were significantly smaller than males (blue) in all islands/islets.
**Figure S3:** Climatic context of the three clustering groups. PCA on 19 bioclimatic variables. Santiago, Brava, Santo Antão, and São Nicolau are represented by multiple points because we sampled at several locations as to represent the environmental diversity within islands.
**Figure S4:** Patterns of phenotypic (P_ST_) and genetic (F_ST_) divergence. P_ST_ was estimated for bill volume, tarsus‐length and wing‐length using trait‐specific heritability (h^2^; value adjacent to trait name) and two values for *C* (0.5. and 1.0). F_ST_ was quantified for the whole‐genome, and genes AXL4 and HMG20B (both associated with bill morphology; likely under selection—Figure 4). Theoretical relationships are depicted: if P_ST_ ≈ F_ST_, neutral processes cannot be excluded as the primary mechanisms for trait variation among populations; by contrast, if P_ST_ is higher or lower than F_ST_, phenotypic differentiation is likely due to diversifying or stabilising selection, respectively.
**Figure S5:** Haplotype sizes for the two chosen genes. (A) Haplotype size for ALX1 and HMG20B genes. The haplotypes of Rombos are significantly bigger than the haplotypes in the West (significances are given after the Wilcoxon test). The Y‐axis gives the size of the region in base pairs. (B) Haplotype size for the ALX1 and HMG20B genes in Rombos vs three random regions along the same chromosome (chromosome 1A for ALX1 and 28 for HMG20B).
**Figure S6:** Dating of haplotypes using tsdate. All choromosomes are labelled and shown. TMRCA: time to the most recent common ancestor.
**Figure S7:** Distribution of time to most recent common ancestor (TMRCA) across the sampled lineages (East, Rombos, and West) in the genomic regions of interest. (A) Boxplots showing the TMRCA distributions within chromosome 1A, where *ALX1* is located (position: 38,465,314–38,471,763 on the chromosome). (B) Boxplots showing the TMRCA distributions for the same populations within chromosome 28, where *HMG20B* is located (positions 825,134‐827,445).
**Figure S8:** Time to most recent common ancestor (TMRCA) for chromosome 1A (A) and 28 (B). Chromosome coordinates are given in the X axis and TMRCA in the Y axis. The vertical red lines highlight the position of *ALX1* in chrmosome 1A and *HMG20B* in chromosome 28.
**Table S1:** Genomic data, including data type (RADseq/WGS), longitude, latitude, island, bird id and species.
**Table S2:** Morphological measurements for 374 adult iago sparrows: 165 females and 209 males.

## Data Availability

All raw sequencing data are available through the European Nucleotide Archive (ENA) under accession number PRJEB108531. All morphological measurements and geographic location data are available at the online Supporting Information.
