# Supplementary material for: Dating the Colonisation and Source of Adaptive Genetic Variation in the Iago Sparrow (Passer iagoensis)
Source: Mol Ecol. 2026 Nov 3;35(21):e70587. doi: 10.1111/mec.70587 (PMC13729259; doi:10.1111/mec.70587)
Supplement: Supplementary file 1 [file MEC-35-e70587-s001.docx]

**Supplementary Figure 1** **Our** **Special K** On the Y axis we plot the error (Evanno-method based) of different clusters (K). The most supported K = 2.

**@ Supplementary Figure 2:** **Sexual dimorphism in the six morphological traits across the entire range of the Iago sparrow**. Females (red) were significantly smaller than males (blue) in all islands/islets.

**Supplementary Figure 3.** **Climatic context of the three clustering groups**. PCA on 19 bioclimatic variables. Santiago, Brava, Santo Antão, and São Nicolau are represented by multiple points because we sampled at several locations as to represent the environmental diversity within islands.

**Supplementary Figure 4.** Patterns of phenotypic (P_ST_) and genetic (F_ST_) divergence. P_ST_ was estimated for bill volume, tarsus-length and wing-length using trait-specific heritability (h^2^; value adjacent to trait name) and two values for *C* (0.5. and 1.0). F_ST_ was quantified for the whole-genome, and genes AXL4 and HMG20B (both associated with bill morphology; likely under selection – Figure 04). Theoretical relationships are depicted: if P_ST_≈F_ST_, neutral processes cannot be excluded as the primary mechanisms for trait variation among populations; by contrast, if P_ST_ is higher or lower than F_ST_, phenotypic differentiation is likely due to diversifying or stabilising selection, respectively.

**Supplementary Figure 5** Haplotype sizes for the two chosen genes. **A - Haplotype size for ALX1 and HMG20B genes**. The haplotypes of Rombos are significantly bigger than the haplotypes in the West (significances are given after the Wilcoxon test). The Y-axis gives the size of the region in base pairs. **B -** Haplotype size for the ALX1 and HMG20B genes in Rombos *vs* three random regions along the same chromosome (chromosome 1A for ALX1 and 28 for HMG20B).

**Supplementary Figure 6**. Dating of haplotypes using tsdate. All choromosomes are labelled and shown. TMRCA: time to the most recent common ancestor.

**Supplementary Figure 7**. Distribution of time to most recent common ancestor (TMRCA) across the sampled lineages (East, Rombos, and West) in the genomic regions of interest. **(A)** Boxplots showing the TMRCA distributions within chromosome 1A, where *ALX1* is located (position: 38,465,314–38,471,763 on the chromosome). **(B)** Boxplots showing the TMRCA distributions for the same populations within chromosome 28, where *HMG20B* is located (positions 825,134-827,445).

A

B

**Supplementary Figure 8.** Time to most recent common ancestor (TMRCA) for chromosome 1A (A) and 28 (B). Chromosome coordinates are given in the X axis and TMRCA in the Y axis. The vertical red lines highlight the position of *ALX1* in chrmosome 1A and *HMG20B* in chromosome 28.

**Supplementary Table 1.** Genomic data, including data type (RADseq/WGS), longitude, latitude, island, bird id and species.

| **Species** | **Bird_ID** | **Latitude** | **Longitude** | **Island** | **RADseq** | **WGS** |
| --- | --- | --- | --- | --- | --- | --- |
| Passer iagoensis | BOA01 | N16º02.781 | W22º41.624 | Boavista | yes | yes |
| Passer iagoensis | BOA02 | N16º02.781 | W22º41.624 | Boavista | yes | - |
| Passer iagoensis | BOA03 | N16º02.781 | W22º41.624 | Boavista | yes | yes |
| Passer iagoensis | BOA04 | N16º02.781 | W22º41.624 | Boavista | yes | - |
| Passer iagoensis | BOA05 | N16º02.781 | W22º41.624 | Boavista | - | - |
| Passer iagoensis | BOA06 | N16º02.781 | W22º41.624 | Boavista | yes | - |
| Passer iagoensis | BOA07 | N16º02.781 | W22º41.624 | Boavista | - | - |
| Passer iagoensis | BOA08 | N16º02.781 | W22º41.624 | Boavista | yes | - |
| Passer iagoensis | BOA09 | N16º02.781 | W22º41.624 | Boavista | - | - |
| Passer iagoensis | BOA10 | N16º02.781 | W22º41.624 | Boavista | - | - |
| Passer iagoensis | BOA11 | N16º02.781 | W22º41.624 | Boavista | - | - |
| Passer iagoensis | BOA12 | N16º02.781 | W22º41.624 | Boavista | - | - |
| Passer iagoensis | BOA13 | N16º02.781 | W22º41.624 | Boavista | - | - |
| Passer iagoensis | BOA14 | N16º02.781 | W22º41.624 | Boavista | - | - |
| Passer iagoensis | BOA15 | N16º02.781 | W22º41.624 | Boavista | yes | - |
| Passer iagoensis | BOA16 | N16º02.781 | W22º41.624 | Boavista | - | - |
| Passer iagoensis | BOA17 | N16º02.781 | W22º41.624 | Boavista | - | - |
| Passer iagoensis | BOA18 | N16º02.781 | W22º41.624 | Boavista | - | - |
| Passer iagoensis | BOA19 | N16º02.781 | W22º41.624 | Boavista | yes | - |
| Passer iagoensis | BOA20 | N16º02.781 | W22º41.624 | Boavista | yes | - |
| Passer iagoensis | BOA21 | N16º02.781 | W22º41.624 | Boavista | yes | - |
| Passer iagoensis | BOA22 | N16º02.781 | W22º41.624 | Boavista | - | - |
| Passer iagoensis | BOA23 | N16º02.781 | W22º41.624 | Boavista | - | - |
| Passer iagoensis | BOA24 | N16º02.781 | W22º41.624 | Boavista | yes | - |
| Passer iagoensis | BOA25 | N16º02.781 | W22º41.624 | Boavista | yes | - |
| Passer iagoensis | BOA26 | N16º02.781 | W22º41.624 | Boavista | - | - |
| Passer iagoensis | BOA27 | N16º02.781 | W22º41.624 | Boavista | yes | - |
| Passer iagoensis | BRA01 | N14º52.249 | W24º41.655 | Brava | - | - |
| Passer iagoensis | BRA02 | N14º52.249 | W24º41.655 | Brava | - | - |
| Passer iagoensis | BRA03 | N14º52.249 | W24º41.655 | Brava | - | - |
| Passer iagoensis | BRA04 | N14º52.249 | W24º41.655 | Brava | - | yes |
| Passer iagoensis | BRA05 | N14º52.249 | W24º41.655 | Brava | - | - |
| Passer iagoensis | BRA08 | N14º52.249 | W24º41.655 | Brava | yes | - |
| Passer iagoensis | BRA09 | N14º52.249 | W24º41.655 | Brava | - | - |
| Passer iagoensis | BRA10 | N14º52.249 | W24º41.655 | Brava | - | - |
| Passer iagoensis | BRA11 | N14º52.249 | W24º41.655 | Brava | - | - |
| Passer iagoensis | BRA12 | N14º52.249 | W24º41.655 | Brava | - | - |
| Passer iagoensis | BRA13 | N14º52.249 | W24º41.655 | Brava | yes | - |
| Passer iagoensis | BRA14 | N14º52.249 | W24º41.655 | Brava | yes | - |
| Passer iagoensis | BRA15 | N14º52.249 | W24º41.655 | Brava | - | - |
| Passer iagoensis | BRA16 | N14º52.249 | W24º41.655 | Brava | yes | - |
| Passer iagoensis | BRA17 | N14º52.249 | W24º41.655 | Brava | yes | - |
| Passer iagoensis | BRA19 | N14º52.249 | W24º41.655 | Brava | yes | - |
| Passer iagoensis | BRA20 | N14º52.249 | W24º41.655 | Brava | yes | - |
| Passer iagoensis | BRA21 | N14º52.249 | W24º41.655 | Brava | - | - |
| Passer iagoensis | BRA23 | N14°52.275 | W24°43.781 | Brava | - | - |
| Passer iagoensis | BRA24 | N14°52.275 | W24°43.781 | Brava | yes | - |
| Passer iagoensis | BRA25 | N14°52.275 | W24°43.781 | Brava | - | - |
| Passer iagoensis | BRA26 | N14°52.275 | W24°43.781 | Brava | yes | - |
| Passer iagoensis | BRA27 | N14°52.275 | W24°43.781 | Brava | yes | yes |
| Passer iagoensis | MAI01 | N15º10.804 | W23º09.195 | Maio | yes | - |
| Passer iagoensis | MAI02 | N15º10.804 | W23º09.195 | Maio | yes | - |
| Passer iagoensis | MAI03 | N15º10.804 | W23º09.195 | Maio | yes | - |
| Passer iagoensis | MAI04 | N15º10.804 | W23º09.195 | Maio | - | - |
| Passer iagoensis | MAI05 | N15º10.804 | W23º09.195 | Maio | - | - |
| Passer iagoensis | MAI06 | N15º10.804 | W23º09.195 | Maio | yes | yes |
| Passer iagoensis | MAI07 | N15º10.804 | W23º09.195 | Maio | yes | - |
| Passer iagoensis | MAI08 | N15º10.804 | W23º09.195 | Maio | yes | - |
| Passer iagoensis | MAI09 | N15º10.804 | W23º09.195 | Maio | - | - |
| Passer iagoensis | MAI10 | N15º10.804 | W23º09.195 | Maio | yes | - |
| Passer iagoensis | MAI11 | N15º10.804 | W23º09.195 | Maio | - | - |
| Passer iagoensis | MAI12 | N15º10.804 | W23º09.195 | Maio | - | - |
| Passer iagoensis | MAI13 | N15º10.804 | W23º09.195 | Maio | yes | - |
| Passer iagoensis | MAI14 | N15º10.804 | W23º09.195 | Maio | yes | - |
| Passer iagoensis | MAI15 | N15º08.626 | W23º07.209 | Maio | - | - |
| Passer iagoensis | MAI16 | N15º08.626 | W23º07.209 | Maio | - | - |
| Passer iagoensis | MAI17 | N15º08.626 | W23º07.209 | Maio | yes | - |
| Passer iagoensis | MAI18 | N15º08.626 | W23º07.209 | Maio | yes | yes |
| Passer iagoensis | MAI19 | N15º08.626 | W23º07.209 | Maio | yes | - |
| Passer iagoensis | MAI20 | N15º08.626 | W23º07.209 | Maio | - | - |
| Passer iagoensis | MAI21 | N15º08.626 | W23º07.209 | Maio | yes | - |
| Passer iagoensis | RBB01 | N14º57.588 | W24º41.659 | Ilheu_Grande | yes | - |
| Passer iagoensis | RBB02 | N14º57.588 | W24º41.659 | Ilheu_Grande | yes | yes |
| Passer iagoensis | RBB03 | N14º57.588 | W24º41.659 | Ilheu_Grande | yes | - |
| Passer iagoensis | RBB04 | N14º57.588 | W24º41.659 | Ilheu_Grande | yes | - |
| Passer iagoensis | RBB05 | N14º57.588 | W24º41.659 | Ilheu_Grande | yes | yes |
| Passer iagoensis | RBB06 | N14º57.588 | W24º41.659 | Ilheu_Grande | yes | yes |
| Passer iagoensis | RBB07 | N14º57.588 | W24º41.659 | Ilheu_Grande | yes | yes |
| Passer iagoensis | RBB08 | N14º57.588 | W24º41.659 | Ilheu_Grande | yes | - |
| Passer iagoensis | RBC01 | N14º58.745 | W24º38.098 | Ilheu_Cima | - | yes |
| Passer iagoensis | RBC02 | N14º58.745 | W24º38.098 | Ilheu_Cima | yes | yes |
| Passer iagoensis | RBC03 | N14º58.745 | W24º38.098 | Ilheu_Cima | - | - |
| Passer iagoensis | RBC04 | N14º58.745 | W24º38.098 | Ilheu_Cima | - | yes |
| Passer iagoensis | RBC05 | N14º58.745 | W24º38.098 | Ilheu_Cima | yes | - |
| Passer iagoensis | RBC06 | N14º58.745 | W24º38.098 | Ilheu_Cima | yes | yes |
| Passer iagoensis | RBC07 | N14º58.745 | W24º38.098 | Ilheu_Cima | - | yes |
| Passer iagoensis | RBC08 | N14º58.745 | W24º38.098 | Ilheu_Cima | - | yes |
| Passer iagoensis | RBC09 | N14º58.745 | W24º38.098 | Ilheu_Cima | - | yes |
| Passer iagoensis | RBC10 | N14º58.745 | W24º38.098 | Ilheu_Cima | - | - |
| Passer iagoensis | RBC11 | N14º58.745 | W24º38.098 | Ilheu_Cima | - | - |
| Passer iagoensis | RBC12 | N14º58.745 | W24º38.098 | Ilheu_Cima | yes | yes |
| Passer iagoensis | RBC13 | N14º58.745 | W24º38.098 | Ilheu_Cima | yes | yes |
| Passer iagoensis | RBC14 | N14º58.745 | W24º38.098 | Ilheu_Cima | - | - |
| Passer iagoensis | RBC15 | N14º58.745 | W24º38.098 | Ilheu_Cima | yes | - |
| Passer iagoensis | RBC16 | N14º58.745 | W24º38.098 | Ilheu_Cima | yes | yes |
| Passer iagoensis | RBC17 | N14º58.745 | W24º38.098 | Ilheu_Cima | - | - |
| Passer iagoensis | RBC18 | N14º58.745 | W24º38.098 | Ilheu_Cima | yes | - |
| Passer iagoensis | RBC19 | N14º58.745 | W24º38.098 | Ilheu_Cima | - | - |
| Passer iagoensis | RBC20 | N14º58.745 | W24º38.098 | Ilheu_Cima | yes | - |
| Passer iagoensis | RBC21 | N14º58.745 | W24º38.098 | Ilheu_Cima | yes | - |
| Passer iagoensis | RBC22 | N14º58.745 | W24º38.098 | Ilheu_Cima | - | - |
| Passer iagoensis | RBC23 | N14º58.745 | W24º38.098 | Ilheu_Cima | - | - |
| Passer iagoensis | RZ01 | N16º36.691 | W24º36.987 | Raso | - | - |
| Passer iagoensis | RZ02 | N16º36.691 | W24º36.987 | Raso | - | - |
| Passer iagoensis | RZ03 | N16º36.691 | W24º36.987 | Raso | - | - |
| Passer iagoensis | RZ04 | N16º36.691 | W24º36.987 | Raso | - | - |
| Passer iagoensis | RZ06 | N16º36.691 | W24º36.987 | Raso | - | - |
| Passer iagoensis | RZ07 | N16º36.691 | W24º36.987 | Raso | - | - |
| Passer iagoensis | RZ08 | N16º36.691 | W24º36.987 | Raso | - | - |
| Passer iagoensis | RZ09 | N16º36.691 | W24º36.987 | Raso | yes | - |
| Passer iagoensis | RZ10 | N16º36.691 | W24º36.987 | Raso | - | - |
| Passer iagoensis | RZ11 | N16º36.691 | W24º36.987 | Raso | - | - |
| Passer iagoensis | RZ12 | N16º36.691 | W24º36.987 | Raso | - | - |
| Passer iagoensis | RZ13 | N16º36.691 | W24º36.987 | Raso | - | - |
| Passer iagoensis | RZ14 | N16º36.691 | W24º36.987 | Raso | - | - |
| Passer iagoensis | RZ15 | N16º36.983 | W24º35.456 | Raso | - | - |
| Passer iagoensis | RZ16 | N16º36.691 | W24º36.987 | Raso | - | yes |
| Passer iagoensis | RZ17 | N16º36.691 | W24º36.987 | Raso | yes | - |
| Passer iagoensis | RZ18 | N16º36.691 | W24º36.987 | Raso | - | - |
| Passer iagoensis | RZ19 | N16º36.691 | W24º36.987 | Raso | yes | - |
| Passer iagoensis | RZ20 | N16º36.691 | W24º36.987 | Raso | - | - |
| Passer iagoensis | RZ21 | N16º36.691 | W24º36.987 | Raso | - | - |
| Passer iagoensis | RZ22 | N16º36.691 | W24º36.987 | Raso | yes | - |
| Passer iagoensis | RZ23 | N16º36.691 | W24º36.987 | Raso | yes | - |
| Passer iagoensis | RZ24 | N16º36.691 | W24º36.987 | Raso | - | - |
| Passer iagoensis | RZ26 | N16º36.691 | W24º36.987 | Raso | yes | - |
| Passer iagoensis | RZ27 | N16º37.216 | W24º34.815 | Raso | - | - |
| Passer iagoensis | RZ28 | N16º37.216 | W24º34.815 | Raso | - | - |
| Passer iagoensis | RZ29 | N16º37.216 | W24º34.815 | Raso | - | - |
| Passer iagoensis | RZ30 | N16º37.216 | W24º34.815 | Raso | yes | - |
| Passer iagoensis | RZ31 | N16º37.216 | W24º34.815 | Raso | yes | - |
| Passer iagoensis | RZ32 | N16º37.216 | W24º34.815 | Raso | yes | - |
| Passer iagoensis | RZ33 | N16º37.184 | W24º34.558 | Raso | - | - |
| Passer iagoensis | RZ34 | N16º37.184 | W24º34.558 | Raso | yes | - |
| Passer iagoensis | RZ35 | N16º37.184 | W24º34.558 | Raso | - | - |
| Passer iagoensis | RZ36 | N16º37.184 | W24º34.558 | Raso | - | - |
| Passer iagoensis | SAL01 | N16º46.642 | W22º56.532 | Sal | - | - |
| Passer iagoensis | SAL02 | N16º46.642 | W22º56.532 | Sal | yes | - |
| Passer iagoensis | SAL03 | N16º46.642 | W22º56.532 | Sal | - | - |
| Passer iagoensis | SAL06 | N16º46.642 | W22º56.532 | Sal | - | - |
| Passer iagoensis | SAL07 | N16º46.642 | W22º56.532 | Sal | yes | - |
| Passer iagoensis | SAL08 | N16º46.642 | W22º56.532 | Sal | yes | yes |
| Passer iagoensis | SAL09 | N16º46.642 | W22º56.532 | Sal | yes | - |
| Passer iagoensis | SAL11 | N16º46.642 | W22º56.532 | Sal | yes | - |
| Passer iagoensis | SAL12 | N16º46.642 | W22º56.532 | Sal | - | - |
| Passer iagoensis | SAL13 | N16º46.642 | W22º56.532 | Sal | - | - |
| Passer iagoensis | SAL14 | N16º46.642 | W22º56.532 | Sal | yes | - |
| Passer iagoensis | SAL16 | N16º46.642 | W22º56.532 | Sal | - | - |
| Passer iagoensis | SAL20 | N16º46.642 | W22º56.532 | Sal | yes | - |
| Passer iagoensis | SAL23 | N16º46.642 | W22º56.532 | Sal | yes | - |
| Passer iagoensis | SAL24 | N16º46.642 | W22º56.532 | Sal | yes | - |
| Passer iagoensis | SAL25 | N16º46.642 | W22º56.532 | Sal | yes | - |
| Passer iagoensis | SAL26 | N16º46.642 | W22º56.532 | Sal | yes | - |
| Passer iagoensis | SAL27 | N16º46.642 | W22º56.532 | Sal | yes | - |
| Passer iagoensis | SAL28 | N16º46.642 | W22º56.532 | Sal | yes | - |
| Passer iagoensis | SAL29 | N16º46.642 | W22º56.532 | Sal | - | - |
| Passer iagoensis | SLZ01 | N16º44.364 | W24º42.758 | Santa_Luzia | yes | - |
| Passer iagoensis | SLZ02 | N16º44.364 | W24º42.758 | Santa_Luzia | yes | - |
| Passer iagoensis | SLZ03 | N16º44.364 | W24º42.758 | Santa_Luzia | - | yes |
| Passer iagoensis | SLZ04 | N16º44.364 | W24º42.758 | Santa_Luzia | yes | - |
| Passer iagoensis | SLZ05 | N16º44.364 | W24º42.758 | Santa_Luzia | - | - |
| Passer iagoensis | SLZ06 | N16º44.364 | W24º42.758 | Santa_Luzia | - | - |
| Passer iagoensis | SLZ07 | N16º44.364 | W24º42.758 | Santa_Luzia | - | - |
| Passer iagoensis | SLZ08 | N16º44.364 | W24º42.758 | Santa_Luzia | - | - |
| Passer iagoensis | SLZ09 | N16º44.364 | W24º42.758 | Santa_Luzia | - | - |
| Passer iagoensis | SLZ10 | N16º44.364 | W24º42.758 | Santa_Luzia | - | - |
| Passer iagoensis | SLZ11 | N16º44.364 | W24º42.758 | Santa_Luzia | - | - |
| Passer iagoensis | SLZ12 | N16°44.770 | W24°44.727 | Santa_Luzia | - | - |
| Passer iagoensis | SLZ15 | N16°44.770 | W24°44.727 | Santa_Luzia | yes | - |
| Passer iagoensis | SLZ16 | N16°44.770 | W24°44.727 | Santa_Luzia | yes | - |
| Passer iagoensis | SLZ17 | N16°44.770 | W24°44.727 | Santa_Luzia | - | - |
| Passer iagoensis | SLZ18 | N16°44.770 | W24°44.727 | Santa_Luzia | - | - |
| Passer iagoensis | SLZ20 | N16°44.770 | W24°44.727 | Santa_Luzia | yes | - |
| Passer iagoensis | SLZ21 | N16°44.770 | W24°44.727 | Santa_Luzia | yes | - |
| Passer iagoensis | SLZ22 | N16°44.770 | W24°44.727 | Santa_Luzia | yes | - |
| Passer iagoensis | SLZ23 | N16°44.770 | W24°44.727 | Santa_Luzia | - | - |
| Passer iagoensis | SLZ24 | N16°44.770 | W24°44.727 | Santa_Luzia | yes | - |
| Passer iagoensis | SLZ25 | N16°44.770 | W24°44.727 | Santa_Luzia | - | - |
| Passer iagoensis | SLZ26 | N16°44.770 | W24°44.727 | Santa_Luzia | yes | - |
| Passer iagoensis | SNI01 | N16°37.245 | W24°20.605 | Sao_Nicolau | yes | - |
| Passer iagoensis | SNI02 | N16°37.245 | W24°20.605 | Sao_Nicolau | yes | - |
| Passer iagoensis | SNI03 | N16°37.245 | W24°20.605 | Sao_Nicolau | yes | - |
| Passer iagoensis | SNI04 | N16°37.245 | W24°20.605 | Sao_Nicolau | yes | - |
| Passer iagoensis | SNI05 | N16°37.245 | W24°20.605 | Sao_Nicolau | yes | yes |
| Passer iagoensis | SNI06 | N16°37.245 | W24°20.605 | Sao_Nicolau | yes | - |
| Passer iagoensis | SNI07 | N16°37.245 | W24°20.605 | Sao_Nicolau | - | - |
| Passer iagoensis | SNI08 | N16°37.245 | W24°20.605 | Sao_Nicolau | yes | - |
| Passer iagoensis | SNI09 | N16°37.245 | W24°20.605 | Sao_Nicolau | yes | - |
| Passer iagoensis | SNI10 | N16°37.245 | W24°20.605 | Sao_Nicolau | yes | - |
| Passer iagoensis | SNI11 | N16°37.245 | W24°20.605 | Sao_Nicolau | yes | - |
| Passer iagoensis | SNI12 | N16°37.245 | W24°20.605 | Sao_Nicolau | yes | - |
| Passer iagoensis | SNI13 | N16°37.245 | W24°20.605 | Sao_Nicolau | yes | - |
| Passer iagoensis | SNI14 | N16°37.245 | W24°20.605 | Sao_Nicolau | yes | - |
| Passer iagoensis | SNI15 | N16°37.245 | W24°20.605 | Sao_Nicolau | - | - |
| Passer iagoensis | SNI18 | N16º34.488 | W24º17.113 | Sao_Nicolau | - | - |
| Passer iagoensis | SNI19 | N16º34.488 | W24º17.113 | Sao_Nicolau | yes | - |
| Passer iagoensis | SNI20 | N16º34.488 | W24º17.113 | Sao_Nicolau | yes | - |
| Passer iagoensis | SNI21 | N16º34.488 | W24º17.113 | Sao_Nicolau | yes | - |
| Passer iagoensis | SNI22 | N16º34.488 | W24º17.113 | Sao_Nicolau | - | - |
| Passer iagoensis | SNI23 | N16º34.488 | W24º17.113 | Sao_Nicolau | yes | - |
| Passer iagoensis | SNI24 | N16º34.488 | W24º17.113 | Sao_Nicolau | yes | - |
| Passer iagoensis | SNI26 | N16º34.488 | W24º17.113 | Sao_Nicolau | yes | - |
| Passer iagoensis | SNI27 | N16º34.488 | W24º17.113 | Sao_Nicolau | yes | - |
| Passer iagoensis | SNI28 | N16º34.488 | W24º17.113 | Sao_Nicolau | - | - |
| Passer iagoensis | SNI29 | N16º34.488 | W24º17.113 | Sao_Nicolau | - | - |
| Passer iagoensis | SNI30 | N16º34.488 | W24º17.113 | Sao_Nicolau | yes | - |
| Passer iagoensis | SNI31 | N16º34.488 | W24º17.113 | Sao_Nicolau | - | - |
| Passer iagoensis | SNI32 | N16º34.488 | W24º17.113 | Sao_Nicolau | - | - |
| Passer iagoensis | SNI33 | N16º34.488 | W24º17.113 | Sao_Nicolau | yes | - |
| Passer iagoensis | SNI34 | N16º34.488 | W24º17.113 | Sao_Nicolau | - | - |
| Passer iagoensis | SNI35 | N16º34.488 | W24º17.113 | Sao_Nicolau | yes | - |
| Passer iagoensis | SNI36 | N16º34.488 | W24º17.113 | Sao_Nicolau | - | - |
| Passer iagoensis | SNI37 | N16º34.488 | W24º17.113 | Sao_Nicolau | - | - |
| Passer iagoensis | SNI38 | N16º34.488 | W24º17.113 | Sao_Nicolau | - | - |
| Passer iagoensis | SNI39 | N16º34.488 | W24º17.113 | Sao_Nicolau | - | - |
| Passer iagoensis | SNI40 | N16º34.488 | W24º17.113 | Sao_Nicolau | yes | - |
| Passer iagoensis | SNI41 | N16º34.488 | W24º17.113 | Sao_Nicolau | - | - |
| Passer iagoensis | SNI42 | N16º34.488 | W24º17.113 | Sao_Nicolau | yes | - |
| Passer iagoensis | SNI43 | N16º34.488 | W24º17.113 | Sao_Nicolau | yes | - |
| Passer iagoensis | SNI44 | N16º35.467 | W24º17.624 | Sao_Nicolau | yes | - |
| Passer iagoensis | SNI45 | N16º35.467 | W24º17.624 | Sao_Nicolau | yes | - |
| Passer iagoensis | SNI46 | N16º35.467 | W24º17.624 | Sao_Nicolau | yes | - |
| Passer iagoensis | SNI47 | N16º35.467 | W24º17.624 | Sao_Nicolau | yes | - |
| Passer iagoensis | SNI48 | N16º35.467 | W24º17.624 | Sao_Nicolau | yes | - |
| Passer iagoensis | SNI49 | N16º35.467 | W24º17.624 | Sao_Nicolau | yes | - |
| Passer iagoensis | SNI51 | N16º35.467 | W24º17.624 | Sao_Nicolau | yes | - |
| Passer iagoensis | SNI52 | N16º35.467 | W24º17.624 | Sao_Nicolau | yes | - |
| Passer iagoensis | SNI53 | N16º35.467 | W24º17.624 | Sao_Nicolau | yes | - |
| Passer iagoensis | SNI54 | N16º35.467 | W24º17.624 | Sao_Nicolau | yes | yes |
| Passer iagoensis | STA01 | N17°07.160 | W25°03.116 | Santo_Antao | yes | - |
| Passer iagoensis | STA02 | N17°07.160 | W25°03.116 | Santo_Antao | yes | - |
| Passer iagoensis | STA03 | N17°07.160 | W25°03.116 | Santo_Antao | yes | - |
| Passer iagoensis | STA04 | N17°07.160 | W25°03.116 | Santo_Antao | - | - |
| Passer iagoensis | STA05 | N17°07.160 | W25°03.116 | Santo_Antao | yes | - |
| Passer iagoensis | STA06 | N17°07.154 | W25°03.098 | Santo_Antao | yes | - |
| Passer iagoensis | STA07 | N17°07.154 | W25°03.098 | Santo_Antao | yes | - |
| Passer iagoensis | STA08 | N17°07.154 | W25°03.098 | Santo_Antao | yes | - |
| Passer iagoensis | STA09 | N17°07.154 | W25°03.098 | Santo_Antao | - | - |
| Passer iagoensis | STA10 | N17°07.154 | W25°03.098 | Santo_Antao | yes | - |
| Passer iagoensis | STA11 | N17°07.154 | W25°03.098 | Santo_Antao | yes | - |
| Passer iagoensis | STA12 | N17°07.154 | W25°03.098 | Santo_Antao | yes | yes |
| Passer iagoensis | STA13 | N17°07.154 | W25°03.098 | Santo_Antao | yes | - |
| Passer iagoensis | STA14 | N17°07.154 | W25°03.098 | Santo_Antao | yes | - |
| Passer iagoensis | STA15 | N17°07.154 | W25°03.098 | Santo_Antao | yes | - |
| Passer iagoensis | STA16 | N17°07.154 | W25°03.098 | Santo_Antao | - | - |
| Passer iagoensis | STA17 | N17°07.154 | W25°03.098 | Santo_Antao | yes | - |
| Passer iagoensis | STA19 | N17°07.154 | W25°03.098 | Santo_Antao | yes | - |
| Passer iagoensis | STA20 | N17°06.916 | W25°03.877 | Santo_Antao | yes | - |
| Passer iagoensis | STA21 | N17°06.916 | W25°03.877 | Santo_Antao | yes | - |
| Passer iagoensis | STA22 | N17°06.916 | W25°03.877 | Santo_Antao | yes | - |
| Passer iagoensis | STA23 | N17°06.916 | W25°03.877 | Santo_Antao | yes | - |
| Passer iagoensis | STA24 | N17°06.916 | W25°03.877 | Santo_Antao | yes | - |
| Passer iagoensis | STA25 | N17°06.916 | W25°03.877 | Santo_Antao | yes | - |
| Passer iagoensis | STA26 | N17°06.916 | W25°03.877 | Santo_Antao | yes | - |
| Passer iagoensis | STA27 | N17°06.916 | W25°03.877 | Santo_Antao | - | - |
| Passer iagoensis | STA28 | N17°06.916 | W25°03.877 | Santo_Antao | - | - |
| Passer iagoensis | STA29 | N17°06.916 | W25°03.877 | Santo_Antao | - | - |
| Passer iagoensis | STA30 | N17°06.916 | W25°03.877 | Santo_Antao | yes | - |
| Passer iagoensis | STA31 | N17°06.916 | W25°03.877 | Santo_Antao | - | - |
| Passer iagoensis | STA32 | N17°06.916 | W25°03.877 | Santo_Antao | - | - |
| Passer iagoensis | STA33 | N17°06.916 | W25°03.877 | Santo_Antao | yes | - |
| Passer iagoensis | STA34 | N17°06.916 | W25°03.877 | Santo_Antao | yes | - |
| Passer iagoensis | STA35 | N17°06.916 | W25°03.877 | Santo_Antao | yes | - |
| Passer iagoensis | STA36 | N17°06.916 | W25°03.877 | Santo_Antao | - | - |
| Passer iagoensis | STA37 | N17°06.916 | W25°03.877 | Santo_Antao | - | - |
| Passer iagoensis | STA38 | N17°06.916 | W25°03.877 | Santo_Antao | yes | - |
| Passer iagoensis | STA39 | N17°06.916 | W25°03.877 | Santo_Antao | - | - |
| Passer iagoensis | STA40 | N17°06.916 | W25°03.877 | Santo_Antao | yes | - |
| Passer iagoensis | STA41 | N17°06.916 | W25°03.877 | Santo_Antao | yes | - |
| Passer iagoensis | STA42 | N17°06.916 | W25°03.877 | Santo_Antao | yes | - |
| Passer iagoensis | STA43 | N16°57.395 | W25°18.627 | Santo_Antao | - | - |
| Passer iagoensis | STA44 | N16°57.395 | W25°18.627 | Santo_Antao | yes | - |
| Passer iagoensis | STA45 | N16°57.395 | W25°18.627 | Santo_Antao | yes | - |
| Passer iagoensis | STA46 | N16°57.395 | W25°18.627 | Santo_Antao | yes | - |
| Passer iagoensis | STA47 | N16°57.395 | W25°18.627 | Santo_Antao | - | - |
| Passer iagoensis | STA48 | N16°57.395 | W25°18.627 | Santo_Antao | - | - |
| Passer iagoensis | STA49 | N16°57.395 | W25°18.627 | Santo_Antao | yes | - |
| Passer iagoensis | STA50 | N16°57.395 | W25°18.627 | Santo_Antao | - | yes |
| Passer iagoensis | STA51 | N16°57.395 | W25°18.627 | Santo_Antao | - | - |
| Passer iagoensis | STA52 | N16°57.395 | W25°18.627 | Santo_Antao | yes | - |
| Passer iagoensis | STA54 | N16°57.395 | W25°18.627 | Santo_Antao | yes | - |
| Passer iagoensis | STA55 | N16°57.395 | W25°18.627 | Santo_Antao | - | - |
| Passer iagoensis | STA56 | N16°57.395 | W25°18.627 | Santo_Antao | yes | - |
| Passer iagoensis | STA57 | N16°57.395 | W25°18.627 | Santo_Antao | yes | - |
| Passer iagoensis | STA58 | N16°57.395 | W25°18.627 | Santo_Antao | yes | - |
| Passer iagoensis | STA59 | N16°57.395 | W25°18.627 | Santo_Antao | yes | - |
| Passer iagoensis | STA60 | N16°57.395 | W25°18.627 | Santo_Antao | yes | - |
| Passer iagoensis | STA61 | N16°57.395 | W25°18.627 | Santo_Antao | yes | - |
| Passer iagoensis | STA62 | N16°57.395 | W25°18.627 | Santo_Antao | - | - |
| Passer iagoensis | STA63 | N16°57.395 | W25°18.627 | Santo_Antao | - | - |
| Passer iagoensis | STA64 | N16°57.395 | W25°18.627 | Santo_Antao | - | - |
| Passer iagoensis | STA65 | N16°57.395 | W25°18.627 | Santo_Antao | yes | - |
| Passer iagoensis | STA66 | N16°57.395 | W25°18.627 | Santo_Antao | - | - |
| Passer iagoensis | STA67 | N16°57.395 | W25°18.627 | Santo_Antao | - | - |
| Passer iagoensis | STA68 | N16°57.395 | W25°18.627 | Santo_Antao | yes | - |
| Passer iagoensis | STA69 | N16°57.395 | W25°18.627 | Santo_Antao | yes | - |
| Passer iagoensis | STG01 | N14°58.390 | W23°27.702 | Santiago | yes | - |
| Passer iagoensis | STG02 | N14°58.390 | W23°27.702 | Santiago | - | yes |
| Passer iagoensis | STG03 | N14°58.390 | W23°27.702 | Santiago | yes | yes |
| Passer iagoensis | STG04 | N14°58.390 | W23°27.702 | Santiago | yes | - |
| Passer iagoensis | STG05 | N14°58.390 | W23°27.702 | Santiago | - | yes |
| Passer iagoensis | STG06 | N14°58.390 | W23°27.702 | Santiago | yes | - |
| Passer iagoensis | STG07 | N14°58.390 | W23°27.702 | Santiago | yes | - |
| Passer iagoensis | STG08 | N14°58.390 | W23°27.702 | Santiago | yes | - |
| Passer iagoensis | STG09 | N14°58.390 | W23°27.702 | Santiago | yes | - |
| Passer iagoensis | STG10 | N14°58.390 | W23°27.702 | Santiago | yes | - |
| Passer iagoensis | STG11 | N14°58.390 | W23°27.702 | Santiago | yes | yes |
| Passer iagoensis | STG12 | N14°58.390 | W23°27.702 | Santiago | yes | - |
| Passer iagoensis | STG13 | N14°58.390 | W23°27.702 | Santiago | - | yes |
| Passer iagoensis | STG14 | N14°58.390 | W23°27.702 | Santiago | yes | - |
| Passer iagoensis | STG15 | N14°58.390 | W23°27.702 | Santiago | yes | - |
| Passer iagoensis | STG16 | N14°58.390 | W23°27.702 | Santiago | - | yes |
| Passer iagoensis | STG17 | N14°58.390 | W23°27.702 | Santiago | yes | - |
| Passer iagoensis | STG18 | N14°58.390 | W23°27.702 | Santiago | - | yes |
| Passer iagoensis | STG19 | N15º16.910 | W23º45.042 | Santiago | yes | - |
| Passer iagoensis | STG20 | N15º16.910 | W23º45.042 | Santiago | - | yes |
| Passer iagoensis | STG21 | N15º16.910 | W23º45.042 | Santiago | yes | - |
| Passer iagoensis | STG25 | N15º16.763 | W23º45.185 | Santiago | yes | - |
| Passer iagoensis | STG26 | N15º16.763 | W23º45.185 | Santiago | yes | yes |
| Passer iagoensis | STG27 | N15º16.763 | W23º45.185 | Santiago | - | - |
| Passer iagoensis | STG29 | N15º16.763 | W23º45.185 | Santiago | - | - |
| Passer iagoensis | STG33 | N15º16.763 | W23º45.185 | Santiago | yes | - |
| Passer iagoensis | STG35 | N15º16.763 | W23º45.185 | Santiago | yes | - |
| Passer iagoensis | STG36 | N15º16.763 | W23º45.185 | Santiago | yes | - |
| Passer iagoensis | STG37 | N15º16.763 | W23º45.185 | Santiago | yes | - |
| Passer iagoensis | STG38 | N15º16.763 | W23º45.185 | Santiago | - | - |
| Passer iagoensis | STG39 | N15º16.763 | W23º45.185 | Santiago | yes | - |
| Passer iagoensis | STG40 | N15º16.763 | W23º45.185 | Santiago | yes | - |
| Passer iagoensis | STG41 | N15º16.763 | W23º45.185 | Santiago | yes | - |
| Passer iagoensis | STG42 | N15º16.763 | W23º45.185 | Santiago | - | - |
| Passer iagoensis | STG43 | N15º16.763 | W23º45.185 | Santiago | - | - |
| Passer iagoensis | STG44 | N15º16.763 | W23º45.185 | Santiago | yes | - |
| Passer iagoensis | STG45 | N15º16.763 | W23º45.185 | Santiago | yes | - |
| Passer iagoensis | STG46 | N15º16.763 | W23º45.185 | Santiago | yes | - |
| Passer iagoensis | STG47 | N15º16.763 | W23º45.185 | Santiago | yes | - |
| Passer iagoensis | STG48 | N15º03.178 | W23º36.309 | Santiago | yes | - |
| Passer iagoensis | STG49 | N15º03.178 | W23º36.309 | Santiago | yes | yes |
| Passer iagoensis | STG50 | N15º03.178 | W23º36.309 | Santiago | yes | - |
| Passer iagoensis | STG51 | N15º03.178 | W23º36.309 | Santiago | yes | - |
| Passer iagoensis | STG52 | N15º03.178 | W23º36.309 | Santiago | - | - |
| Passer iagoensis | STG53 | N15º03.178 | W23º36.309 | Santiago | yes | - |
| Passer iagoensis | STG56 | N15º03.178 | W23º36.309 | Santiago | yes | - |
| Passer iagoensis | STG57 | N15º03.178 | W23º36.309 | Santiago | yes | - |
| Passer iagoensis | STG58 | N15º03.178 | W23º36.309 | Santiago | - | - |
| Passer iagoensis | STG59 | N15°03.340 | W23°36.122 | Santiago | yes | - |
| Passer iagoensis | STG60 | N15°03.048 | W23°36.529 | Santiago | yes | - |
| Passer iagoensis | STG61 | N15°03.048 | W23°36.529 | Santiago | - | - |
| Passer iagoensis | STG62 | N15°03.048 | W23°36.529 | Santiago | - | - |
| Passer iagoensis | STG63 | N15°03.048 | W23°36.529 | Santiago | yes | - |
| Passer iagoensis | STG64 | N15°03.048 | W23°36.529 | Santiago | yes | - |
| Passer iagoensis | STG65 | N15°03.048 | W23°36.529 | Santiago | - | - |
| Passer iagoensis | STG66 | N15°03.048 | W23°36.529 | Santiago | yes | - |
| Passer iagoensis | STG68 | N15°03.048 | W23°36.529 | Santiago | - | - |
| Passer iagoensis | STG69 | N15°03.048 | W23°36.529 | Santiago | yes | - |
| Passer iagoensis | STG70 | N15°03.048 | W23°36.529 | Santiago | - | - |
| Passer iagoensis | STG71 | N15°03.048 | W23°36.529 | Santiago | - | - |
| Passer iagoensis | STG72 | N15°03.048 | W23°36.529 | Santiago | - | - |
| Passer iagoensis | STG73 | N14°58.390 | W23°27.702 | Santiago | yes | - |
| Passer iagoensis | STG74 | N14°58.390 | W23°27.702 | Santiago | yes | - |
| Passer iagoensis | STG75 | N14°58.390 | W23°27.702 | Santiago | - | - |
| Passer iagoensis | SV01 | N16°51.674 | W24°56.261 | Sao_Vicente | yes | - |
| Passer iagoensis | SV02 | N16°51.674 | W24°56.261 | Sao_Vicente | - | - |
| Passer iagoensis | SV03 | N16°51.674 | W24°56.261 | Sao_Vicente | yes | yes |
| Passer iagoensis | SV04 | N16°51.674 | W24°56.261 | Sao_Vicente | yes | - |
| Passer iagoensis | SV05 | N16°51.674 | W24°56.261 | Sao_Vicente | - | - |
| Passer iagoensis | SV06 | N16°51.674 | W24°56.261 | Sao_Vicente | - | - |
| Passer iagoensis | SV07 | N16°51.674 | W24°56.261 | Sao_Vicente | yes | - |
| Passer iagoensis | SV09 | N16°51.674 | W24°56.261 | Sao_Vicente | yes | - |
| Passer iagoensis | SV10 | N16°51.674 | W24°56.261 | Sao_Vicente | yes | - |
| Passer iagoensis | SV11 | N16°51.674 | W24°56.261 | Sao_Vicente | yes | - |
| Passer iagoensis | SV12 | N16°51.674 | W24°56.261 | Sao_Vicente | yes | - |
| Passer iagoensis | SV14 | N16°51.674 | W24°56.261 | Sao_Vicente | yes | - |
| Passer iagoensis | SV15 | N16°51.674 | W24°56.261 | Sao_Vicente | yes | - |
| Passer iagoensis | SV16 | N16°51.674 | W24°56.261 | Sao_Vicente | - | - |
| Passer iagoensis | SV17 | N16°51.674 | W24°56.261 | Sao_Vicente | yes | - |
| Passer iagoensis | SV18 | N16°51.674 | W24°56.261 | Sao_Vicente | - | - |
| Passer iagoensis | SV19 | N16°51.674 | W24°56.261 | Sao_Vicente | yes | - |
| Passer iagoensis | SV20 | N16°51.674 | W24°56.261 | Sao_Vicente | yes | - |

**Supplementary Table 2. Morphological measurements** for 374 adult iago sparrows: 165 females and 209 males.

| **Species** | **Bird_ID** | **Latitude** | **Longitude** | **Island** | **Wing_Length** | **Tarsus-length** | **Bill_length** | **Bill_width** | **Bill_depth** | **Sex** |
| --- | --- | --- | --- | --- | --- | --- | --- | --- | --- | --- |
| Passer iagoensis | BOA02 | N16º02.781 | W22º41.624 | Boavista | 60,0 | 16,17 | 8,93 | 6,74 | 7,59 | F |
| Passer iagoensis | BOA04 | N16º02.781 | W22º41.624 | Boavista | 60,0 | 17,16 | 8,95 | 6,43 | 7,01 | F |
| Passer iagoensis | BOA08 | N16º02.781 | W22º41.624 | Boavista | 59,5 | 17,10 | 8,93 | 6,51 | 7,43 | F |
| Passer iagoensis | BOA09 | N16º02.781 | W22º41.624 | Boavista | 60,5 | 18,32 | 8,96 | 6,65 | 7,85 | F |
| Passer iagoensis | BOA12 | N16º02.781 | W22º41.624 | Boavista | 58,0 | 17,52 | 8,84 | 6,62 | 7,40 | F |
| Passer iagoensis | BOA17 | N16º02.781 | W22º41.624 | Boavista | 59,0 | 17,74 | 9,16 | 6,63 | 7,19 | F |
| Passer iagoensis | BOA19 | N16º02.781 | W22º41.624 | Boavista | 60,0 | 17,59 | 9,11 | 6,73 | 7,24 | F |
| Passer iagoensis | BOA20 | N16º02.781 | W22º41.624 | Boavista | 61,0 | 17,73 | 8,71 | 6,60 | 7,42 | F |
| Passer iagoensis | BOA21 | N16º02.781 | W22º41.624 | Boavista | 60,0 | 17,32 | 8,89 | 6,75 | 7,50 | F |
| Passer iagoensis | BOA23 | N16º02.781 | W22º41.624 | Boavista | 58,0 | 16,91 | 8,80 | 6,17 | 7,37 | F |
| Passer iagoensis | BOA26 | N16º02.781 | W22º41.624 | Boavista | 60,5 | 17,40 | 8,69 | 6,79 | 7,47 | F |
| Passer iagoensis | BRA23 | N14°52.275 | W24°43.781 | Brava | 61,5 | 19,08 | 9,54 | 6,52 | 7,48 | F |
| Passer iagoensis | BRA24 | N14°52.275 | W24°43.781 | Brava | 59,0 | 17,60 | 8,92 | 6,58 | 6,98 | F |
| Passer iagoensis | BRA26 | N14°52.275 | W24°43.781 | Brava | 61,0 | 18,06 | 9,15 | 6,85 | 7,30 | F |
| Passer iagoensis | BRA01 | N14º52.249 | W24º41.655 | Brava | 62,0 | 17,48 | 8,71 | 6,45 | 7,15 | F |
| Passer iagoensis | BRA02 | N14º52.249 | W24º41.655 | Brava | 60,0 | 18,44 | 8,77 | 6,32 | 7,15 | F |
| Passer iagoensis | BRA09 | N14º52.249 | W24º41.655 | Brava | 61,0 | 18,61 | 8,56 | 6,32 | 7,27 | F |
| Passer iagoensis | BRA10 | N14º52.249 | W24º41.655 | Brava | 59,0 | 19,32 | 9,55 | 7,18 | 7,65 | F |
| Passer iagoensis | BRA12 | N14º52.249 | W24º41.655 | Brava | 61,0 | 17,93 | 8,82 | 6,20 | 7,05 | F |
| Passer iagoensis | BRA13 | N14º52.249 | W24º41.655 | Brava | 62,0 | 18,90 | 8,95 | 6,81 | 7,49 | F |
| Passer iagoensis | BRA14 | N14º52.249 | W24º41.655 | Brava | 62,0 | 19,14 | 8,75 | 6,78 | 7,62 | F |
| Passer iagoensis | BRA17 | N14º52.249 | W24º41.655 | Brava | 63,0 | 19,43 | 9,07 | 6,91 | 7,51 | F |
| Passer iagoensis | BRA21 | N14º52.249 | W24º41.655 | Brava | 60,0 | 19,68 | 8,70 | 6,46 | 7,52 | F |
| Passer iagoensis | MAI01 | N15º10.804 | W23º09.195 | Maio | 60,0 | 16,76 | 8,96 | 6,46 | 7,17 | F |
| Passer iagoensis | MAI02 | N15º10.804 | W23º09.195 | Maio | 59,0 | 16,41 | 8,93 | 6,31 | 7,42 | F |
| Passer iagoensis | MAI04 | N15º10.804 | W23º09.195 | Maio | 59,5 | 17,25 | 8,58 | 6,31 | 7,74 | F |
| Passer iagoensis | MAI05 | N15º10.804 | W23º09.195 | Maio | 57,0 | 18,16 | 9,52 | 6,74 | 7,58 | F |
| Passer iagoensis | MAI07 | N15º10.804 | W23º09.195 | Maio | 62,0 | 17,82 | 9,16 | 6,88 | 8,04 | F |
| Passer iagoensis | MAI09 | N15º10.804 | W23º09.195 | Maio | 59,0 | 17,31 | 8,72 | 6,54 | 7,69 | F |
| Passer iagoensis | MAI11 | N15º10.804 | W23º09.195 | Maio | 59,0 | 17,56 | 8,69 | 6,84 | 7,56 | F |
| Passer iagoensis | MAI13 | N15º10.804 | W23º09.195 | Maio | 58,0 | 16,92 | 8,38 | 6,19 | 7,47 | F |
| Passer iagoensis | MAI15 | N15º08.626 | W23º07.209 | Maio | 59,5 | 15,59 | 9,07 | 6,41 | 7,56 | F |
| Passer iagoensis | MAI16 | N15º08.626 | W23º07.209 | Maio | 63,0 | 18,32 | 9,84 | 6,05 | 7,40 | F |
| Passer iagoensis | MAI17 | N15º08.626 | W23º07.209 | Maio | 61,0 | 16,89 | 9,55 | 6,73 | 7,49 | F |
| Passer iagoensis | MAI21 | N15º08.626 | W23º07.209 | Maio | 58,0 | 16,60 | 8,78 | 6,50 | 7,50 | F |
| Passer iagoensis | RZ07 | N16º36.691 | W24º36.987 | Raso | 63,0 | 17,61 | 8,53 | 6,49 | 7,14 | F |
| Passer iagoensis | RZ08 | N16º36.691 | W24º36.987 | Raso | 59,0 | 17,18 | 8,48 | 6,32 | 7,40 | F |
| Passer iagoensis | RZ09 | N16º36.691 | W24º36.987 | Raso | 60,0 | 16,92 | 9,03 | 6,38 | 7,16 | F |
| Passer iagoensis | RZ11 | N16º36.691 | W24º36.987 | Raso | 59,0 | 17,49 | 8,45 | 6,07 | 7,18 | F |
| Passer iagoensis | RZ12 | N16º36.691 | W24º36.987 | Raso | 63,0 | 17,63 | 9,19 | 6,73 | 7,36 | F |
| Passer iagoensis | RZ13 | N16º36.691 | W24º36.987 | Raso | 61,0 | 17,48 | 8,68 | 6,33 | 7,07 | F |
| Passer iagoensis | RZ14 | N16º36.691 | W24º36.987 | Raso | 60,0 | 17,96 | 8,92 | 6,40 | 7,23 | F |
| Passer iagoensis | RZ15 | N16º36.983 | W24º35.456 | Raso | 56,5 | 16,02 | 8,28 | 6,41 | 6,75 | F |
| Passer iagoensis | RZ17 | N16º36.691 | W24º36.987 | Raso | 61,0 | 17,82 | 8,00 | 6,12 | 7,19 | F |
| Passer iagoensis | RZ19 | N16º36.691 | W24º36.987 | Raso | 61,0 | 17,35 | 8,54 | 6,71 | 7,37 | F |
| Passer iagoensis | RZ20 | N16º36.691 | W24º36.987 | Raso | 62,0 | 17,30 | 8,78 | 6,76 | 7,05 | F |
| Passer iagoensis | RZ26 | N16º36.691 | W24º36.987 | Raso | 59,5 | 17,45 | 8,66 | 6,25 | 7,43 | F |
| Passer iagoensis | RZ27 | N16º37.216 | W24º34.815 | Raso | 59,0 | 17,36 | 8,38 | 6,14 | 7,07 | F |
| Passer iagoensis | RZ32 | N16º37.216 | W24º34.815 | Raso | 59,5 | 17,64 | 8,16 | 6,08 | 7,02 | F |
| Passer iagoensis | RZ33 | N16º37.184 | W24º34.558 | Raso | 60,0 | 17,78 | 8,45 | 6,16 | 6,97 | F |
| Passer iagoensis | RBB01 | N14º57.588 | W24º41.659 | Ilheu_Grande | 65,0 | 19,03 | 9,50 | 6,82 | 8,18 | F |
| Passer iagoensis | RBB05 | N14º57.588 | W24º41.659 | Ilheu_Grande | 64,0 | 18,72 | 9,18 | 7,07 | 7,88 | F |
| Passer iagoensis | RBB06 | N14º57.588 | W24º41.659 | Ilheu_Grande | 62,0 | 19,46 | 10,03 | 6,75 | 8,38 | F |
| Passer iagoensis | RBB08 | N14º57.588 | W24º41.659 | Ilheu_Grande | 62,0 | 18,78 | 9,24 | 6,68 | 8,30 | F |
| Passer iagoensis | RBC01 | N14º58.745 | W24º38.098 | Ilheu_Cima | 64,0 | 19,35 | 9,28 | 6,85 | 7,84 | F |
| Passer iagoensis | RBC05 | N14º58.745 | W24º38.098 | Ilheu_Cima | 63,0 | 17,97 | 9,33 | 6,75 | 7,79 | F |
| Passer iagoensis | RBC07 | N14º58.745 | W24º38.098 | Ilheu_Cima | 68,0 | 18,73 | 9,54 | 6,90 | 7,90 | F |
| Passer iagoensis | RBC11 | N14º58.745 | W24º38.098 | Ilheu_Cima | 65,0 | 18,69 | 9,36 | 6,68 | 8,40 | F |
| Passer iagoensis | RBC12 | N14º58.745 | W24º38.098 | Ilheu_Cima | 53,0 | 18,07 | 9,38 | 6,88 | 7,90 | F |
| Passer iagoensis | RBC13 | N14º58.745 | W24º38.098 | Ilheu_Cima | 62,0 | 18,09 | 12,42 | 6,73 | 7,73 | F |
| Passer iagoensis | RBC15 | N14º58.745 | W24º38.098 | Ilheu_Cima | 64,5 | 19,25 | 9,87 | 7,08 | 8,06 | F |
| Passer iagoensis | RBC19 | N14º58.745 | W24º38.098 | Ilheu_Cima | 63,0 | 19,31 | 9,56 | 7,01 | 8,19 | F |
| Passer iagoensis | RBC20 | N14º58.745 | W24º38.098 | Ilheu_Cima | 64,0 | 18,48 | 9,41 | 7,07 | 8,23 | F |
| Passer iagoensis | RBC22 | N14º58.745 | W24º38.098 | Ilheu_Cima | 63,0 | 17,74 | 9,91 | 6,39 | 8,00 | F |
| Passer iagoensis | RBC23 | N14º58.745 | W24º38.098 | Ilheu_Cima | 62,0 | 18,77 | 10,54 | 6,76 | 7,52 | F |
| Passer iagoensis | SAL01 | N16º46.642 | W22º56.532 | Sal | 58,5 | 17,96 | 8,70 | 6,60 | 7,69 | F |
| Passer iagoensis | SAL02 | N16º46.642 | W22º56.532 | Sal | 58,0 | 17,19 | 8,59 | 6,36 | 7,60 | F |
| Passer iagoensis | SAL11 | N16º46.642 | W22º56.532 | Sal | 58,0 | 16,91 | 8,85 | 6,40 | 7,16 | F |
| Passer iagoensis | SAL14 | N16º46.642 | W22º56.532 | Sal | 58,5 | 17,58 | 9,18 | 6,34 | 7,60 | F |
| Passer iagoensis | SAL25 | N16º46.642 | W22º56.532 | Sal | 60,0 | 17,48 | 8,94 | 6,78 | 7,68 | F |
| Passer iagoensis | SAL26 | N16º46.642 | W22º56.532 | Sal | 61,0 | 17,38 | 9,21 | 6,24 | 7,31 | F |
| Passer iagoensis | SAL28 | N16º46.642 | W22º56.532 | Sal | 60,0 | 17,64 | 9,32 | 6,62 | 7,26 | F |
| Passer iagoensis | SLZ01 | N16º44.364 | W24º42.758 | Santa_Luzia | 60,0 | 18,06 | 8,48 | 6,25 | 6,94 | F |
| Passer iagoensis | SLZ04 | N16º44.364 | W24º42.758 | Santa_Luzia | 58,0 | 17,22 | 7,96 | 6,84 | 6,92 | F |
| Passer iagoensis | SLZ12 | N16°44.770 | W24°44.727 | Santa_Luzia | 59,0 | 17,97 | 8,65 | 5,90 | 7,33 | F |
| Passer iagoensis | SLZ15 | N16°44.770 | W24°44.727 | Santa_Luzia | 57,0 | 17,13 | 8,58 | 6,15 | 7,23 | F |
| Passer iagoensis | SLZ17 | N16°44.770 | W24°44.727 | Santa_Luzia | 59,0 | 16,82 | 8,38 | 6,01 | 6,99 | F |
| Passer iagoensis | SLZ18 | N16°44.770 | W24°44.727 | Santa_Luzia | 56,5 | 17,91 | 8,45 | 6,03 | 6,95 | F |
| Passer iagoensis | SLZ19 | N16°44.770 | W24°44.727 | Santa_Luzia | 60,0 | 17,96 | 9,00 | 6,16 | 7,18 | F |
| Passer iagoensis | SLZ21 | N16°44.770 | W24°44.727 | Santa_Luzia | 57,0 | 17,31 | 8,35 | 5,98 | 6,95 | F |
| Passer iagoensis | STG02 | N14°58.390 | W23°27.702 | Santiago | 60,0 | 16,55 | 9,61 | 6,76 | 7,61 | F |
| Passer iagoensis | STG03 | N14°58.390 | W23°27.702 | Santiago | 59,0 | 16,93 | 8,94 | 6,84 | 7,77 | F |
| Passer iagoensis | STG04 | N14°58.390 | W23°27.702 | Santiago | 59,0 | 17,05 | 8,66 | 6,55 | 7,34 | F |
| Passer iagoensis | STG05 | N14°58.390 | W23°27.702 | Santiago | 61,5 | 17,73 | 9,91 | 7,37 | 8,17 | F |
| Passer iagoensis | STG06 | N14°58.390 | W23°27.702 | Santiago | 61,0 | 17,09 | 9,12 | 6,57 | 7,68 | F |
| Passer iagoensis | STG09 | N14°58.390 | W23°27.702 | Santiago | 61,0 | 17,11 | 8,43 | 6,15 | 7,54 | F |
| Passer iagoensis | STG12 | N14°58.390 | W23°27.702 | Santiago | 60,0 | 16,15 | 9,23 | 6,95 | 7,31 | F |
| Passer iagoensis | STG13 | N14°58.390 | W23°27.702 | Santiago | 59,0 | 17,35 | 9,16 | 6,70 | 7,75 | F |
| Passer iagoensis | STG17 | N14°58.390 | W23°27.702 | Santiago | 58,0 | 16,05 | 8,99 | 6,28 | 7,19 | F |
| Passer iagoensis | STG48 | N15º03.178 | W23º36.309 | Santiago | 61,0 | 16,51 | 9,62 | 6,54 | 7,14 | F |
| Passer iagoensis | STG52 | N15º03.178 | W23º36.309 | Santiago | 61,0 | 16,86 | 9,34 | 6,35 | 7,11 | F |
| Passer iagoensis | STG57 | N15º03.178 | W23º36.309 | Santiago | 61,0 | 17,58 | 10,05 | 6,63 | 7,68 | F |
| Passer iagoensis | STG59 | N15°03.340 | W23°36.122 | Santiago | 59,0 | 17,63 | 9,59 | 6,45 | 7,87 | F |
| Passer iagoensis | STG60 | N15°03.048 | W23°36.529 | Santiago | 60,0 | 16,62 | 9,46 | 6,29 | 7,18 | F |
| Passer iagoensis | STG62 | N15°03.048 | W23°36.529 | Santiago | 60,0 | 17,28 | 9,47 | 6,47 | 7,83 | F |
| Passer iagoensis | STG63 | N15°03.048 | W23°36.529 | Santiago | 60,5 | 17,71 | 9,70 | 6,64 | 7,41 | F |
| Passer iagoensis | STG68 | N15°03.048 | W23°36.529 | Santiago | 60,0 | 17,07 | 9,83 | 6,67 | 7,96 | F |
| Passer iagoensis | STG69 | N15°03.048 | W23°36.529 | Santiago | 63,0 | 17,46 | 9,60 | 6,78 | 7,41 | F |
| Passer iagoensis | STG71 | N15°03.048 | W23°36.529 | Santiago | 63,0 | 17,80 | 9,79 | 6,78 | 7,61 | F |
| Passer iagoensis | STG72 | N15°03.048 | W23°36.529 | Santiago | 61,0 | 17,99 | 9,24 | 6,20 | 7,13 | F |
| Passer iagoensis | STG27 | N15º16.763 | W23º45.185 | Santiago | 60,0 | 15,58 | 8,91 | 6,16 | 7,52 | F |
| Passer iagoensis | STG29 | N15º16.763 | W23º45.185 | Santiago | 60,0 | 16,70 | 9,03 | 6,45 | 7,12 | F |
| Passer iagoensis | STG33 | N15º16.763 | W23º45.185 | Santiago | 56,0 | 16,31 | 8,91 | 6,28 | 7,51 | F |
| Passer iagoensis | STG37 | N15º16.763 | W23º45.185 | Santiago | 64,0 | 16,59 | 8,92 | 6,19 | 7,65 | F |
| Passer iagoensis | STG38 | N15º16.763 | W23º45.185 | Santiago | 60,0 | 16,38 | 9,77 | 6,22 | 7,49 | F |
| Passer iagoensis | STG39 | N15º16.763 | W23º45.185 | Santiago | 60,0 | 17,31 | 9,04 | 6,61 | 7,34 | F |
| Passer iagoensis | STG40 | N15º16.763 | W23º45.185 | Santiago | 60,0 | 16,86 | 9,28 | 6,33 | 7,66 | F |
| Passer iagoensis | STG41 | N15º16.763 | W23º45.185 | Santiago | 61,0 | 17,20 | 8,59 | 6,19 | 7,39 | F |
| Passer iagoensis | STG42 | N15º16.763 | W23º45.185 | Santiago | 60,0 | 16,88 | 8,52 | 6,43 | 7,31 | F |
| Passer iagoensis | STG43 | N15º16.763 | W23º45.185 | Santiago | 64,0 | 17,36 | 8,76 | 6,19 | 7,41 | F |
| Passer iagoensis | STG47 | N15º16.763 | W23º45.185 | Santiago | 59,0 | 17,74 | 9,37 | 6,77 | 7,51 | F |
| Passer iagoensis | STG19 | N15º16.910 | W23º45.042 | Santiago | 58,0 | 16,72 | 8,63 | 6,26 | 7,26 | F |
| Passer iagoensis | STG20 | N15º16.910 | W23º45.042 | Santiago | 60,0 | 16,32 | 8,69 | 6,48 | 7,84 | F |
| Passer iagoensis | STA03 | N17°07.160 | W25°03.116 | Santo_Antao | 57,0 | 17,37 | 8,33 | 6,81 | 7,21 | F |
| Passer iagoensis | STA06 | N17°07.154 | W25°03.098 | Santo_Antao | 60,0 | 17,19 | 8,38 | 6,03 | 7,16 | F |
| Passer iagoensis | STA10 | N17°07.154 | W25°03.098 | Santo_Antao | 63,0 | 18,61 | 8,40 | 6,73 | 7,05 | F |
| Passer iagoensis | STA16 | N17°07.154 | W25°03.098 | Santo_Antao | 59,0 | 16,90 | 8,64 | 6,72 | 7,18 | F |
| Passer iagoensis | STA19 | N17°07.154 | W25°03.098 | Santo_Antao | 57,0 | 16,95 | 8,49 | 6,35 | 7,04 | F |
| Passer iagoensis | STA20 | N17°06.916 | W25°03.877 | Santo_Antao | 59,5 | 17,00 | 8,28 | 6,25 | 6,90 | F |
| Passer iagoensis | STA24 | N17°06.916 | W25°03.877 | Santo_Antao | 57,5 | 16,73 | 7,83 | 6,42 | 6,62 | F |
| Passer iagoensis | STA25 | N17°06.916 | W25°03.877 | Santo_Antao | 59,0 | 17,36 | 8,18 | 6,17 | 7,16 | F |
| Passer iagoensis | STA26 | N17°06.916 | W25°03.877 | Santo_Antao | 61,0 | 15,80 | 8,57 | 6,28 | 7,49 | F |
| Passer iagoensis | STA39 | N17°06.916 | W25°03.877 | Santo_Antao | 57,0 | 17,61 | 8,66 | 6,09 | 7,18 | F |
| Passer iagoensis | STA40 | N17°06.916 | W25°03.877 | Santo_Antao | 60,0 | 16,94 | 8,19 | 6,26 | 7,03 | F |
| Passer iagoensis | STA41 | N17°06.916 | W25°03.877 | Santo_Antao | 60,0 | 18,20 | 8,58 | 6,63 | 7,11 | F |
| Passer iagoensis | STA42 | N17°06.916 | W25°03.877 | Santo_Antao | 61,5 | 17,28 | 8,45 | 6,82 | 7,42 | F |
| Passer iagoensis | STA43 | N16°57.395 | W25°18.627 | Santo_Antao | 62,0 | 17,59 | 9,31 | 6,27 | 7,34 | F |
| Passer iagoensis | STA45 | N16°57.395 | W25°18.627 | Santo_Antao | 60,0 | 17,18 | 9,16 | 6,53 | 7,22 | F |
| Passer iagoensis | STA47 | N16°57.395 | W25°18.627 | Santo_Antao | 59,0 | 16,52 | 8,79 | 6,35 | 7,35 | F |
| Passer iagoensis | STA49 | N16°57.395 | W25°18.627 | Santo_Antao | 58,0 | 16,47 | 8,00 | 6,84 | 7,12 | F |
| Passer iagoensis | STA55 | N16°57.395 | W25°18.627 | Santo_Antao | 58,5 | 16,77 | 9,00 | 6,59 | 7,25 | F |
| Passer iagoensis | STA57 | N16°57.395 | W25°18.627 | Santo_Antao | 60,0 | 16,78 | 8,66 | 6,67 | 7,24 | F |
| Passer iagoensis | STA58 | N16°57.395 | W25°18.627 | Santo_Antao | 59,0 | 17,07 | 7,92 | 6,54 | 6,76 | F |
| Passer iagoensis | STA60 | N16°57.395 | W25°18.627 | Santo_Antao | 60,0 | 17,17 | 8,63 | 6,40 | 7,13 | F |
| Passer iagoensis | STA61 | N16°57.395 | W25°18.627 | Santo_Antao | 59,0 | 17,73 | 8,57 | 6,58 | 7,06 | F |
| Passer iagoensis | STA62 | N16°57.395 | W25°18.627 | Santo_Antao | 60,0 | 17,71 | 8,32 | 6,95 | 7,42 | F |
| Passer iagoensis | STA63 | N16°57.395 | W25°18.627 | Santo_Antao | 60,0 | 17,27 | 8,49 | 6,56 | 7,12 | F |
| Passer iagoensis | STA64 | N16°57.395 | W25°18.627 | Santo_Antao | 59,0 | 17,60 | 8,33 | 6,03 | 6,80 | F |
| Passer iagoensis | STA67 | N16°57.395 | W25°18.627 | Santo_Antao | 61,0 | 17,89 | 8,41 | 6,59 | 7,17 | F |
| Passer iagoensis | STA68 | N16°57.395 | W25°18.627 | Santo_Antao | 59,5 | 17,39 | 9,40 | 6,48 | 7,57 | F |
| Passer iagoensis | SNI44 | N16º35.467 | W24º17.624 | Sao_Nicolau | 57,0 | 16,46 | 9,31 | 7,02 | 7,58 | F |
| Passer iagoensis | SNI45 | N16º35.467 | W24º17.624 | Sao_Nicolau | 59,0 | 17,11 | 8,98 | 6,22 | 7,41 | F |
| Passer iagoensis | SNI47 | N16º35.467 | W24º17.624 | Sao_Nicolau | 60,0 | 16,97 | 8,38 | 6,87 | 7,05 | F |
| Passer iagoensis | SNI49 | N16º35.467 | W24º17.624 | Sao_Nicolau | 59,0 | 18,08 | 8,75 | 6,58 | 7,06 | F |
| Passer iagoensis | SNI54 | N16º35.467 | W24º17.624 | Sao_Nicolau | 60,0 | 17,42 | 8,63 | 6,51 | 7,31 | F |
| Passer iagoensis | SNI03 | N16°37.245 | W24°20.605 | Sao_Nicolau | 62,0 | 18,07 | 8,91 | 6,70 | 7,51 | F |
| Passer iagoensis | SNI04 | N16°37.245 | W24°20.605 | Sao_Nicolau | 61,0 | 17,78 | 9,07 | 6,21 | 7,23 | F |
| Passer iagoensis | SNI10 | N16°37.245 | W24°20.605 | Sao_Nicolau | 60,0 | 17,06 | 8,39 | 6,72 | 7,61 | F |
| Passer iagoensis | SNI11 | N16°37.245 | W24°20.605 | Sao_Nicolau | 64,0 | 17,79 | 8,95 | 6,41 | 7,56 | F |
| Passer iagoensis | SNI13 | N16°37.245 | W24°20.605 | Sao_Nicolau | 61,0 | 16,80 | 9,45 | 6,98 | 7,65 | F |
| Passer iagoensis | SNI14 | N16°37.245 | W24°20.605 | Sao_Nicolau | 60,0 | 17,36 | 8,33 | 5,91 | 6,60 | F |
| Passer iagoensis | SNI19 | N16º34.488 | W24º17.113 | Sao_Nicolau | 62,0 | 17,52 | 8,89 | 6,36 | 7,48 | F |
| Passer iagoensis | SNI21 | N16º34.488 | W24º17.113 | Sao_Nicolau | 61,0 | 16,99 | 8,86 | 6,67 | 7,12 | F |
| Passer iagoensis | SNI26 | N16º34.488 | W24º17.113 | Sao_Nicolau | 60,0 | 17,73 | 8,50 | 6,27 | 7,11 | F |
| Passer iagoensis | SNI27 | N16º34.488 | W24º17.113 | Sao_Nicolau | 58,0 | 16,48 | 9,03 | 6,30 | 6,99 | F |
| Passer iagoensis | SNI38 | N16º34.488 | W24º17.113 | Sao_Nicolau | 61,0 | 17,47 | 8,99 | 6,51 | 7,28 | F |
| Passer iagoensis | SNI39 | N16º34.488 | W24º17.113 | Sao_Nicolau | 58,0 | 16,71 | 8,74 | 6,55 | 7,19 | F |
| Passer iagoensis | SNI40 | N16º34.488 | W24º17.113 | Sao_Nicolau | 60,0 | 17,70 | 8,11 | 6,43 | 7,33 | F |
| Passer iagoensis | SNI41 | N16º34.488 | W24º17.113 | Sao_Nicolau | 57,0 | 16,40 | 8,88 | 6,16 | 7,12 | F |
| Passer iagoensis | SNI42 | N16º34.488 | W24º17.113 | Sao_Nicolau | 61,0 | 18,30 | 9,24 | 6,71 | 7,44 | F |
| Passer iagoensis | SNI43 | N16º34.488 | W24º17.113 | Sao_Nicolau | 61,0 | 17,68 | 8,53 | 6,50 | 7,25 | F |
| Passer iagoensis | SV09 | N16°51.674 | W24°56.261 | Sao_Vicente | 62,0 | 17,81 | 8,09 | 6,45 | 6,90 | F |
| Passer iagoensis | SV11 | N16°51.674 | W24°56.261 | Sao_Vicente | 60,0 | 17,81 | 8,85 | 6,90 | 7,20 | F |
| Passer iagoensis | SV12 | N16°51.674 | W24°56.261 | Sao_Vicente | 60,0 | 17,14 | 8,32 | 6,05 | 7,04 | F |
| Passer iagoensis | SV14 | N16°51.674 | W24°56.261 | Sao_Vicente | 60,0 | 17,29 | 8,84 | 5,95 | 7,31 | F |
| Passer iagoensis | BOA01 | N16º02.781 | W22º41.624 | Boavista | 65,0 | 18,12 | 10,06 | 7,46 | 8,15 | M |
| Passer iagoensis | BOA03 | N16º02.781 | W22º41.624 | Boavista | 64,5 | 17,07 | 10,50 | 7,19 | 8,04 | M |
| Passer iagoensis | BOA05 | N16º02.781 | W22º41.624 | Boavista | 65,0 | 18,23 | 10,01 | 7,38 | 8,03 | M |
| Passer iagoensis | BOA06 | N16º02.781 | W22º41.624 | Boavista | 66,0 | 18,57 | 10,33 | 7,25 | 8,59 | M |
| Passer iagoensis | BOA07 | N16º02.781 | W22º41.624 | Boavista | 65,0 | 17,89 | 10,06 | 7,08 | 8,16 | M |
| Passer iagoensis | BOA10 | N16º02.781 | W22º41.624 | Boavista | 64,0 | 18,10 | 10,01 | 6,75 | 7,86 | M |
| Passer iagoensis | BOA11 | N16º02.781 | W22º41.624 | Boavista | 63,0 | 17,78 | 9,92 | 7,23 | 8,23 | M |
| Passer iagoensis | BOA13 | N16º02.781 | W22º41.624 | Boavista | 66,5 | 18,10 | 10,02 | 7,40 | 8,41 | M |
| Passer iagoensis | BOA14 | N16º02.781 | W22º41.624 | Boavista | 63,5 | 17,37 | 9,88 | 7,00 | 8,09 | M |
| Passer iagoensis | BOA15 | N16º02.781 | W22º41.624 | Boavista | 64,0 | 17,83 | 9,77 | 7,07 | 7,82 | M |
| Passer iagoensis | BOA16 | N16º02.781 | W22º41.624 | Boavista | 66,0 | 18,70 | 10,47 | 7,41 | 8,69 | M |
| Passer iagoensis | BOA18 | N16º02.781 | W22º41.624 | Boavista | 66,0 | 18,59 | 10,01 | 7,71 | 8,60 | M |
| Passer iagoensis | BOA22 | N16º02.781 | W22º41.624 | Boavista | 68,0 | 19,13 | 10,10 | 6,92 | 8,16 | M |
| Passer iagoensis | BOA24 | N16º02.781 | W22º41.624 | Boavista | 65,5 | 18,32 | 9,85 | 7,15 | 8,54 | M |
| Passer iagoensis | BOA25 | N16º02.781 | W22º41.624 | Boavista | 66,0 | 18,51 | 10,09 | 7,12 | 8,67 | M |
| Passer iagoensis | BOA27 | N16º02.781 | W22º41.624 | Boavista | 65,5 | 18,09 | 10,25 | 7,52 | 8,63 | M |
| Passer iagoensis | BRA25 | N14°52.275 | W24°43.781 | Brava | 66,0 | 19,13 | 10,52 | 7,61 | 8,51 | M |
| Passer iagoensis | BRA27 | N14°52.275 | W24°43.781 | Brava | 67,0 | 20,47 | 10,50 | 7,17 | 8,58 | M |
| Passer iagoensis | BRA03 | N14º52.249 | W24º41.655 | Brava | 66,0 | 20,08 | 9,59 | 7,45 | 8,10 | M |
| Passer iagoensis | BRA05 | N14º52.249 | W24º41.655 | Brava | 65,0 | 19,24 | 10,61 | 7,28 | 8,44 | M |
| Passer iagoensis | BRA08 | N14º52.249 | W24º41.655 | Brava | 66,0 | 19,55 | 10,03 | 7,27 | 8,82 | M |
| Passer iagoensis | BRA11 | N14º52.249 | W24º41.655 | Brava | 67,5 | 19,78 | 10,70 | 7,54 | 8,63 | M |
| Passer iagoensis | BRA15 | N14º52.249 | W24º41.655 | Brava | 64,0 | 20,49 | 9,79 | 7,54 | 8,10 | M |
| Passer iagoensis | BRA16 | N14º52.249 | W24º41.655 | Brava | 68,0 | 20,41 | 10,43 | 7,38 | 8,16 | M |
| Passer iagoensis | BRA19 | N14º52.249 | W24º41.655 | Brava | 65,0 | 19,59 | 10,04 | 7,27 | 8,28 | M |
| Passer iagoensis | BRA20 | N14º52.249 | W24º41.655 | Brava | 65,0 | 19,22 | 10,64 | 7,59 | 8,76 | M |
| Passer iagoensis | MAI03 | N15º10.804 | W23º09.195 | Maio | 65,0 | 18,54 | 10,51 | 7,28 | 8,66 | M |
| Passer iagoensis | MAI06 | N15º10.804 | W23º09.195 | Maio | 67,0 | 18,03 | 10,93 | 7,63 | 8,83 | M |
| Passer iagoensis | MAI08 | N15º10.804 | W23º09.195 | Maio | 64,0 | 18,64 | 9,65 | 7,34 | 8,41 | M |
| Passer iagoensis | MAI10 | N15º10.804 | W23º09.195 | Maio | 65,0 | 18,68 | 9,84 | 7,27 | 8,60 | M |
| Passer iagoensis | MAI12 | N15º10.804 | W23º09.195 | Maio | 64,0 | 19,13 | 10,60 | 7,39 | 8,71 | M |
| Passer iagoensis | MAI14 | N15º10.804 | W23º09.195 | Maio | 65,0 | 18,80 | 10,50 | 7,24 | 8,88 | M |
| Passer iagoensis | MAI18 | N15º08.626 | W23º07.209 | Maio | 66,0 | 18,26 | 9,92 | 7,45 | 8,48 | M |
| Passer iagoensis | MAI19 | N15º08.626 | W23º07.209 | Maio | 65,0 | 18,83 | 10,40 | 7,43 | 8,42 | M |
| Passer iagoensis | MAI20 | N15º08.626 | W23º07.209 | Maio | 64,5 | 18,35 | 9,95 | 7,12 | 8,88 | M |
| Passer iagoensis | RZ01 | N16º36.691 | W24º36.987 | Raso | 64,0 | 18,32 | 9,64 | 7,13 | 8,01 | M |
| Passer iagoensis | RZ02 | N16º36.691 | W24º36.987 | Raso | 67,0 | 18,60 | 10,46 | 7,28 | 8,53 | M |
| Passer iagoensis | RZ03 | N16º36.691 | W24º36.987 | Raso | 67,0 | 18,60 | 10,06 | 7,51 | 8,52 | M |
| Passer iagoensis | RZ04 | N16º36.691 | W24º36.987 | Raso | 67,5 | 18,95 | 10,32 | 7,23 | 8,40 | M |
| Passer iagoensis | RZ06 | N16º36.691 | W24º36.987 | Raso | 65,0 | 18,80 | 9,44 | 7,24 | 8,10 | M |
| Passer iagoensis | RZ10 | N16º36.691 | W24º36.987 | Raso | 63,0 | 18,10 | 9,95 | 7,00 | 8,02 | M |
| Passer iagoensis | RZ16 | N16º36.691 | W24º36.987 | Raso | 65,0 | 18,47 | 9,70 | 6,99 | 8,41 | M |
| Passer iagoensis | RZ18 | N16º36.691 | W24º36.987 | Raso | 65,0 | 18,53 | 9,22 | 6,74 | 8,34 | M |
| Passer iagoensis | RZ21 | N16º36.691 | W24º36.987 | Raso | 65,0 | 18,08 | 9,61 | 7,08 | 8,29 | M |
| Passer iagoensis | RZ22 | N16º36.691 | W24º36.987 | Raso | 64,0 | 18,25 | 9,66 | 6,99 | 8,11 | M |
| Passer iagoensis | RZ23 | N16º36.691 | W24º36.987 | Raso | 66,5 | 18,92 | 10,46 | 7,35 | 8,68 | M |
| Passer iagoensis | RZ24 | N16º36.691 | W24º36.987 | Raso | 67,0 | 18,40 | 9,43 | 6,78 | 8,09 | M |
| Passer iagoensis | RZ28 | N16º37.216 | W24º34.815 | Raso | 67,0 | 18,20 | 10,18 | 6,99 | 8,41 | M |
| Passer iagoensis | RZ29 | N16º37.216 | W24º34.815 | Raso | 63,0 | 18,56 | 9,52 | 7,12 | 7,93 | M |
| Passer iagoensis | RZ30 | N16º37.216 | W24º34.815 | Raso | 63,5 | 17,92 | 9,17 | 6,81 | 7,72 | M |
| Passer iagoensis | RZ31 | N16º37.216 | W24º34.815 | Raso | 63,0 | 19,23 | 9,22 | 6,63 | 8,12 | M |
| Passer iagoensis | RZ34 | N16º37.184 | W24º34.558 | Raso | 65,0 | 18,59 | 9,80 | 7,29 | 8,33 | M |
| Passer iagoensis | RZ35 | N16º37.184 | W24º34.558 | Raso | 62,0 | 18,17 | 9,64 | 6,42 | 8,38 | M |
| Passer iagoensis | RZ36 | N16º37.184 | W24º34.558 | Raso | 66,0 | 18,92 | 9,96 | 7,25 | 8,60 | M |
| Passer iagoensis | RBB02 | N14º57.588 | W24º41.659 | Ilheu_Grande | 70,0 | 20,31 | 11,64 | 7,71 | 9,28 | M |
| Passer iagoensis | RBB03 | N14º57.588 | W24º41.659 | Ilheu_Grande | 67,0 | 19,71 | 10,69 | 7,50 | 9,14 | M |
| Passer iagoensis | RBB04 | N14º57.588 | W24º41.659 | Ilheu_Grande | 70,0 | 19,34 | 10,59 | 7,57 | 8,92 | M |
| Passer iagoensis | RBB07 | N14º57.588 | W24º41.659 | Ilheu_Grande | 67,0 | 19,97 | 11,21 | 7,61 | 9,58 | M |
| Passer iagoensis | RBC02 | N14º58.745 | W24º38.098 | Ilheu_Cima | 69,5 | 20,09 | 10,91 | 7,72 | 8,87 | M |
| Passer iagoensis | RBC03 | N14º58.745 | W24º38.098 | Ilheu_Cima | 70,0 | 19,86 | 10,34 | 7,72 | 9,43 | M |
| Passer iagoensis | RBC04 | N14º58.745 | W24º38.098 | Ilheu_Cima | 70,0 | 20,00 | 10,60 | 7,53 | 9,42 | M |
| Passer iagoensis | RBC06 | N14º58.745 | W24º38.098 | Ilheu_Cima | 68,0 | 20,21 | 11,13 | 7,90 | 9,22 | M |
| Passer iagoensis | RBC08 | N14º58.745 | W24º38.098 | Ilheu_Cima | 72,0 | 20,20 | 10,58 | 7,61 | 9,06 | M |
| Passer iagoensis | RBC09 | N14º58.745 | W24º38.098 | Ilheu_Cima | 69,5 | 19,26 | 10,86 | 7,25 | 9,26 | M |
| Passer iagoensis | RBC10 | N14º58.745 | W24º38.098 | Ilheu_Cima | 66,0 | 19,17 | 10,58 | 7,70 | 8,99 | M |
| Passer iagoensis | RBC14 | N14º58.745 | W24º38.098 | Ilheu_Cima | 67,5 | 19,26 | 10,07 | 7,40 | 8,85 | M |
| Passer iagoensis | RBC16 | N14º58.745 | W24º38.098 | Ilheu_Cima | 67,0 | 19,06 | 10,33 | 7,23 | 8,88 | M |
| Passer iagoensis | RBC17 | N14º58.745 | W24º38.098 | Ilheu_Cima | 70,5 | 18,83 | 10,56 | 7,17 | 9,35 | M |
| Passer iagoensis | RBC18 | N14º58.745 | W24º38.098 | Ilheu_Cima | 67,0 | 19,31 | 10,25 | 7,44 | 9,22 | M |
| Passer iagoensis | RBC21 | N14º58.745 | W24º38.098 | Ilheu_Cima | 68,0 | 19,48 | 10,71 | 7,65 | 8,96 | M |
| Passer iagoensis | SAL03 | N16º46.642 | W22º56.532 | Sal | 65,0 | 18,52 | 10,08 | 6,87 | 8,32 | M |
| Passer iagoensis | SAL06 | N16º46.642 | W22º56.532 | Sal | 67,5 | 18,64 | 10,64 | 7,81 | 8,79 | M |
| Passer iagoensis | SAL07 | N16º46.642 | W22º56.532 | Sal | 66,0 | 18,72 | 10,30 | 7,12 | 8,38 | M |
| Passer iagoensis | SAL08 | N16º46.642 | W22º56.532 | Sal | 61,0 | 18,37 | 10,11 | 7,15 | 8,07 | M |
| Passer iagoensis | SAL09 | N16º46.642 | W22º56.532 | Sal | 65,0 | 18,64 | 10,01 | 7,54 | 8,65 | M |
| Passer iagoensis | SAL12 | N16º46.642 | W22º56.532 | Sal | 63,0 | 17,92 | 10,95 | 7,22 | 8,50 | M |
| Passer iagoensis | SAL13 | N16º46.642 | W22º56.532 | Sal | 63,5 | 18,52 | 9,71 | 7,30 | 8,50 | M |
| Passer iagoensis | SAL16 | N16º46.642 | W22º56.532 | Sal | 64,0 | 18,86 | 10,02 | 7,10 | 8,25 | M |
| Passer iagoensis | SAL20 | N16º46.642 | W22º56.532 | Sal | 64,0 | 18,99 | 10,58 | 6,99 | 8,68 | M |
| Passer iagoensis | SAL23 | N16º46.642 | W22º56.532 | Sal | 65,0 | 18,45 | 11,03 | 6,98 | 8,28 | M |
| Passer iagoensis | SAL24 | N16º46.642 | W22º56.532 | Sal | 64,0 | 18,61 | 9,92 | 7,20 | 8,61 | M |
| Passer iagoensis | SAL27 | N16º46.642 | W22º56.532 | Sal | 66,0 | 18,34 | 10,59 | 7,57 | 9,03 | M |
| Passer iagoensis | SAL29 | N16º46.642 | W22º56.532 | Sal | 65,0 | 18,21 | 10,07 | 7,35 | 8,52 | M |
| Passer iagoensis | SLZ02 | N16º44.364 | W24º42.758 | Santa_Luzia | 65,0 | 18,44 | 9,79 | 6,98 | 8,23 | M |
| Passer iagoensis | SLZ03 | N16º44.364 | W24º42.758 | Santa_Luzia | 64,5 | 19,02 | 10,18 | 7,02 | 8,39 | M |
| Passer iagoensis | SLZ05 | N16º44.364 | W24º42.758 | Santa_Luzia | 65,0 | 18,24 | 9,39 | 6,85 | 8,24 | M |
| Passer iagoensis | SLZ06 | N16º44.364 | W24º42.758 | Santa_Luzia | 53,5 | 18,50 | 9,85 | 6,23 | 8,15 | M |
| Passer iagoensis | SLZ07 | N16º44.364 | W24º42.758 | Santa_Luzia | 64,5 | 19,01 | 9,75 | 7,17 | 7,95 | M |
| Passer iagoensis | SLZ08 | N16º44.364 | W24º42.758 | Santa_Luzia | 64,0 | 18,91 | 9,92 | 7,16 | 7,75 | M |
| Passer iagoensis | SLZ09 | N16º44.364 | W24º42.758 | Santa_Luzia | 65,0 | 18,40 | 9,58 | 6,50 | 8,08 | M |
| Passer iagoensis | SLZ10 | N16º44.364 | W24º42.758 | Santa_Luzia | 63,0 | 18,68 | 10,45 | 6,73 | 8,53 | M |
| Passer iagoensis | SLZ11 | N16º44.364 | W24º42.758 | Santa_Luzia | 63,0 | 18,74 | 9,80 | 7,27 | 8,12 | M |
| Passer iagoensis | SLZ16 | N16°44.770 | W24°44.727 | Santa_Luzia | 61,0 | 18,53 | 9,56 | 7,06 | 7,98 | M |
| Passer iagoensis | SLZ22 | N16°44.770 | W24°44.727 | Santa_Luzia | 65,0 | 17,93 | 9,66 | 6,85 | 7,60 | M |
| Passer iagoensis | SLZ23 | N16°44.770 | W24°44.727 | Santa_Luzia | 65,0 | 18,95 | 9,83 | 6,93 | 7,86 | M |
| Passer iagoensis | SLZ24 | N16°44.770 | W24°44.727 | Santa_Luzia | 63,0 | 18,78 | 10,37 | 7,13 | 8,00 | M |
| Passer iagoensis | SLZ25 | N16°44.770 | W24°44.727 | Santa_Luzia | 62,0 | 17,70 | 10,00 | 6,57 | 8,01 | M |
| Passer iagoensis | SLZ26 | N16°44.770 | W24°44.727 | Santa_Luzia | 63,0 | 18,76 | 9,65 | 6,85 | 8,36 | M |
| Passer iagoensis | STG01 | N14°58.390 | W23°27.702 | Santiago | 62,0 | 18,15 | 10,53 | 7,32 | 8,70 | M |
| Passer iagoensis | STG07 | N14°58.390 | W23°27.702 | Santiago | 65,0 | 18,81 | 10,03 | 6,95 | 8,23 | M |
| Passer iagoensis | STG08 | N14°58.390 | W23°27.702 | Santiago | 65,0 | 17,94 | 10,37 | 7,48 | 8,24 | M |
| Passer iagoensis | STG10 | N14°58.390 | W23°27.702 | Santiago | 64,0 | 18,59 | 10,26 | 7,40 | 8,51 | M |
| Passer iagoensis | STG11 | N14°58.390 | W23°27.702 | Santiago | 65,0 | 15,87 | 9,62 | 6,95 | 7,64 | M |
| Passer iagoensis | STG14 | N14°58.390 | W23°27.702 | Santiago | 66,0 | 18,79 | 10,60 | 7,58 | 8,89 | M |
| Passer iagoensis | STG15 | N14°58.390 | W23°27.702 | Santiago | 64,0 | 18,81 | 10,53 | 7,09 | 8,30 | M |
| Passer iagoensis | STG16 | N14°58.390 | W23°27.702 | Santiago | 63,0 | 18,66 | 10,44 | 7,50 | 8,59 | M |
| Passer iagoensis | STG73 | N14°58.390 | W23°27.702 | Santiago | 63,0 | 17,92 | 9,88 | 7,66 | 8,48 | M |
| Passer iagoensis | STG74 | N14°58.390 | W23°27.702 | Santiago | 65,0 | 18,78 | 10,21 | 6,91 | 8,13 | M |
| Passer iagoensis | STG75 | N14°58.390 | W23°27.702 | Santiago | 65,0 | 17,18 | 10,60 | 7,56 | 8,67 | M |
| Passer iagoensis | STG49 | N15º03.178 | W23º36.309 | Santiago | 64,0 | 18,67 | 10,31 | 7,27 | 8,39 | M |
| Passer iagoensis | STG50 | N15º03.178 | W23º36.309 | Santiago | 65,0 | 19,17 | 11,06 | 7,47 | 8,56 | M |
| Passer iagoensis | STG51 | N15º03.178 | W23º36.309 | Santiago | 67,0 | 18,99 | 10,95 | 7,78 | 8,64 | M |
| Passer iagoensis | STG53 | N15º03.178 | W23º36.309 | Santiago | 63,0 | 18,33 | 10,71 | 7,20 | 8,52 | M |
| Passer iagoensis | STG56 | N15º03.178 | W23º36.309 | Santiago | 67,0 | 18,18 | 10,97 | 7,51 | 8,38 | M |
| Passer iagoensis | STG58 | N15º03.178 | W23º36.309 | Santiago | 64,0 | 18,39 | 10,82 | 7,11 | 8,29 | M |
| Passer iagoensis | STG61 | N15°03.048 | W23°36.529 | Santiago | 65,0 | 18,17 | 10,47 | 7,08 | 8,58 | M |
| Passer iagoensis | STG64 | N15°03.048 | W23°36.529 | Santiago | 64,0 | 19,14 | 11,49 | 7,74 | 8,69 | M |
| Passer iagoensis | STG65 | N15°03.048 | W23°36.529 | Santiago | 65,0 | 18,79 | 10,76 | 7,55 | 8,53 | M |
| Passer iagoensis | STG66 | N15°03.048 | W23°36.529 | Santiago | 68,0 | 17,75 | 9,81 | 7,00 | 8,34 | M |
| Passer iagoensis | STG70 | N15°03.048 | W23°36.529 | Santiago | 67,0 | 18,08 | 10,63 | 7,45 | 8,59 | M |
| Passer iagoensis | STG25 | N15º16.763 | W23º45.185 | Santiago | 64,0 | 17,73 | 10,22 | 6,82 | 8,56 | M |
| Passer iagoensis | STG26 | N15º16.763 | W23º45.185 | Santiago | 64,0 | 18,37 | 10,58 | 7,13 | 8,51 | M |
| Passer iagoensis | STG35 | N15º16.763 | W23º45.185 | Santiago | 67,0 | 18,49 | 10,36 | 7,17 | 8,47 | M |
| Passer iagoensis | STG36 | N15º16.763 | W23º45.185 | Santiago | 58,0 | 17,97 | 10,21 | 6,78 | 8,61 | M |
| Passer iagoensis | STG44 | N15º16.763 | W23º45.185 | Santiago | 66,0 | 18,77 | 9,94 | 7,62 | 8,41 | M |
| Passer iagoensis | STG45 | N15º16.763 | W23º45.185 | Santiago | 64,0 | 17,22 | 10,45 | 7,64 | 8,84 | M |
| Passer iagoensis | STG46 | N15º16.763 | W23º45.185 | Santiago | 65,0 | 18,78 | 10,55 | 6,95 | 8,18 | M |
| Passer iagoensis | STG21 | N15º16.910 | W23º45.042 | Santiago | 64,0 | 18,08 | 9,88 | 7,08 | 8,56 | M |
| Passer iagoensis | STA01 | N17°07.160 | W25°03.116 | Santo_Antao | 65,5 | 18,39 | 9,76 | 7,26 | 7,98 | M |
| Passer iagoensis | STA02 | N17°07.160 | W25°03.116 | Santo_Antao | 62,0 | 17,49 | 9,91 | 7,29 | 7,50 | M |
| Passer iagoensis | STA04 | N17°07.160 | W25°03.116 | Santo_Antao | 63,5 | 19,22 | 9,93 | 7,30 | 8,22 | M |
| Passer iagoensis | STA05 | N17°07.160 | W25°03.116 | Santo_Antao | 67,0 | 19,44 | 9,90 | 7,47 | 8,34 | M |
| Passer iagoensis | STA08 | N17°07.154 | W25°03.098 | Santo_Antao | 63,0 | 18,54 | 9,29 | 6,93 | 8,33 | M |
| Passer iagoensis | STA09 | N17°07.154 | W25°03.098 | Santo_Antao | 66,0 | 19,12 | 10,19 | 7,07 | 8,11 | M |
| Passer iagoensis | STA11 | N17°07.154 | W25°03.098 | Santo_Antao | 65,0 | 18,54 | 9,52 | 7,23 | 7,80 | M |
| Passer iagoensis | STA12 | N17°07.154 | W25°03.098 | Santo_Antao | 66,0 | 18,89 | 9,76 | 7,33 | 8,28 | M |
| Passer iagoensis | STA13 | N17°07.154 | W25°03.098 | Santo_Antao | 65,5 | 18,76 | 9,89 | 7,26 | 7,82 | M |
| Passer iagoensis | STA14 | N17°07.154 | W25°03.098 | Santo_Antao | 64,0 | 17,81 | 9,56 | 7,02 | 7,81 | M |
| Passer iagoensis | STA15 | N17°07.154 | W25°03.098 | Santo_Antao | 65,0 | 19,06 | 10,07 | 7,46 | 8,06 | M |
| Passer iagoensis | STA17 | N17°07.154 | W25°03.098 | Santo_Antao | 65,0 | 18,22 | 9,21 | 6,75 | 7,64 | M |
| Passer iagoensis | STA21 | N17°06.916 | W25°03.877 | Santo_Antao | 66,0 | 18,22 | 9,67 | 6,99 | 7,91 | M |
| Passer iagoensis | STA22 | N17°06.916 | W25°03.877 | Santo_Antao | 65,0 | 17,29 | 9,69 | 6,72 | 7,74 | M |
| Passer iagoensis | STA23 | N17°06.916 | W25°03.877 | Santo_Antao | 65,5 | 18,31 | 9,69 | 6,87 | 8,01 | M |
| Passer iagoensis | STA27 | N17°06.916 | W25°03.877 | Santo_Antao | 65,0 | 17,95 | 9,05 | 7,20 | 7,88 | M |
| Passer iagoensis | STA28 | N17°06.916 | W25°03.877 | Santo_Antao | 63,0 | 19,11 | 9,63 | 6,97 | 7,37 | M |
| Passer iagoensis | STA29 | N17°06.916 | W25°03.877 | Santo_Antao | 67,0 | 17,61 | 9,44 | 7,22 | 8,02 | M |
| Passer iagoensis | STA30 | N17°06.916 | W25°03.877 | Santo_Antao | 65,0 | 17,57 | 9,61 | 7,09 | 7,65 | M |
| Passer iagoensis | STA31 | N17°06.916 | W25°03.877 | Santo_Antao | 64,0 | 18,70 | 9,30 | 6,86 | 7,99 | M |
| Passer iagoensis | STA32 | N17°06.916 | W25°03.877 | Santo_Antao | 64,0 | 17,89 | 9,73 | 6,81 | 8,03 | M |
| Passer iagoensis | STA33 | N17°06.916 | W25°03.877 | Santo_Antao | 67,0 | 16,79 | 8,94 | 6,86 | 7,49 | M |
| Passer iagoensis | STA34 | N17°06.916 | W25°03.877 | Santo_Antao | 64,0 | 18,68 | 10,08 | 7,34 | 8,25 | M |
| Passer iagoensis | STA35 | N17°06.916 | W25°03.877 | Santo_Antao | 63,0 | 17,54 | 9,59 | 6,68 | 7,48 | M |
| Passer iagoensis | STA36 | N17°06.916 | W25°03.877 | Santo_Antao | 62,0 | 17,65 | 9,21 | 6,72 | 7,79 | M |
| Passer iagoensis | STA37 | N17°06.916 | W25°03.877 | Santo_Antao | 68,0 | 18,63 | 9,39 | 7,06 | 7,78 | M |
| Passer iagoensis | STA38 | N17°06.916 | W25°03.877 | Santo_Antao | 64,0 | 18,32 | 10,00 | 6,69 | 7,79 | M |
| Passer iagoensis | STA44 | N16°57.395 | W25°18.627 | Santo_Antao | 63,0 | 17,84 | 9,99 | 7,25 | 7,94 | M |
| Passer iagoensis | STA46 | N16°57.395 | W25°18.627 | Santo_Antao | 62,0 | 18,81 | 10,00 | 7,41 | 8,33 | M |
| Passer iagoensis | STA48 | N16°57.395 | W25°18.627 | Santo_Antao | 62,0 | 17,45 | 9,78 | 6,98 | 8,21 | M |
| Passer iagoensis | STA50 | N16°57.395 | W25°18.627 | Santo_Antao | 64,0 | 18,70 | 9,27 | 7,25 | 7,87 | M |
| Passer iagoensis | STA51 | N16°57.395 | W25°18.627 | Santo_Antao | 63,5 | 18,48 | 9,90 | 7,03 | 8,10 | M |
| Passer iagoensis | STA52 | N16°57.395 | W25°18.627 | Santo_Antao | 64,0 | 17,10 | 9,26 | 7,10 | 7,72 | M |
| Passer iagoensis | STA54 | N16°57.395 | W25°18.627 | Santo_Antao | 66,5 | 17,73 | 9,54 | 6,90 | 7,90 | M |
| Passer iagoensis | STA56 | N16°57.395 | W25°18.627 | Santo_Antao | 64,0 | 17,97 | 9,08 | 7,27 | 8,10 | M |
| Passer iagoensis | STA59 | N16°57.395 | W25°18.627 | Santo_Antao | 63,0 | 18,56 | 9,27 | 6,81 | 7,82 | M |
| Passer iagoensis | STA65 | N16°57.395 | W25°18.627 | Santo_Antao | 64,0 | 18,01 | 9,82 | 7,19 | 7,81 | M |
| Passer iagoensis | STA66 | N16°57.395 | W25°18.627 | Santo_Antao | 65,0 | 18,11 | 9,57 | 7,25 | 8,16 | M |
| Passer iagoensis | STA69 | N16°57.395 | W25°18.627 | Santo_Antao | 64,5 | 18,30 | 10,02 | 6,90 | 7,78 | M |
| Passer iagoensis | SNI46 | N16º35.467 | W24º17.624 | Sao_Nicolau | 63,0 | 17,63 | 9,54 | 6,63 | 7,87 | M |
| Passer iagoensis | SNI48 | N16º35.467 | W24º17.624 | Sao_Nicolau | 65,0 | 18,39 | 9,62 | 6,84 | 8,13 | M |
| Passer iagoensis | SNI51 | N16º35.467 | W24º17.624 | Sao_Nicolau | 61,0 | 18,64 | 6,91 | 7,84 | 7,89 | M |
| Passer iagoensis | SNI52 | N16º35.467 | W24º17.624 | Sao_Nicolau | 60,0 | 17,21 | 6,92 | 6,86 | 7,74 | M |
| Passer iagoensis | SNI01 | N16°37.245 | W24°20.605 | Sao_Nicolau | 63,5 | 17,68 | 10,09 | 6,97 | 8,22 | M |
| Passer iagoensis | SNI02 | N16°37.245 | W24°20.605 | Sao_Nicolau | 66,5 | 19,00 | 10,10 | 7,64 | 8,46 | M |
| Passer iagoensis | SNI05 | N16°37.245 | W24°20.605 | Sao_Nicolau | 66,0 | 18,66 | 9,99 | 7,17 | 8,79 | M |
| Passer iagoensis | SNI06 | N16°37.245 | W24°20.605 | Sao_Nicolau | 66,0 | 18,96 | 10,47 | 7,33 | 8,30 | M |
| Passer iagoensis | SNI07 | N16°37.245 | W24°20.605 | Sao_Nicolau | 65,0 | 19,31 | 10,41 | 7,19 | 8,76 | M |
| Passer iagoensis | SNI08 | N16°37.245 | W24°20.605 | Sao_Nicolau | 65,0 | 18,74 | 10,39 | 7,29 | 8,16 | M |
| Passer iagoensis | SNI09 | N16°37.245 | W24°20.605 | Sao_Nicolau | 66,0 | 19,08 | 10,78 | 7,18 | 8,28 | M |
| Passer iagoensis | SNI12 | N16°37.245 | W24°20.605 | Sao_Nicolau | 64,5 | 17,61 | 9,46 | 7,00 | 7,54 | M |
| Passer iagoensis | SNI15 | N16°37.245 | W24°20.605 | Sao_Nicolau | 65,0 | 18,76 | 10,82 | 7,77 | 7,39 | M |
| Passer iagoensis | SNI18 | N16º34.488 | W24º17.113 | Sao_Nicolau | 63,0 | 16,91 | 9,59 | 6,99 | 8,14 | M |
| Passer iagoensis | SNI20 | N16º34.488 | W24º17.113 | Sao_Nicolau | 62,0 | 17,56 | 9,67 | 7,12 | 7,67 | M |
| Passer iagoensis | SNI22 | N16º34.488 | W24º17.113 | Sao_Nicolau | 64,5 | 18,45 | 9,39 | 6,78 | 8,39 | M |
| Passer iagoensis | SNI23 | N16º34.488 | W24º17.113 | Sao_Nicolau | 65,0 | 19,80 | 9,79 | 7,38 | 8,48 | M |
| Passer iagoensis | SNI24 | N16º34.488 | W24º17.113 | Sao_Nicolau | 66,0 | 18,20 | 9,42 | 7,21 | 8,65 | M |
| Passer iagoensis | SNI28 | N16º34.488 | W24º17.113 | Sao_Nicolau | 65,0 | 18,34 | 10,24 | 7,28 | 8,28 | M |
| Passer iagoensis | SNI29 | N16º34.488 | W24º17.113 | Sao_Nicolau | 64,0 | 18,26 | 9,78 | 7,24 | 8,15 | M |
| Passer iagoensis | SNI30 | N16º34.488 | W24º17.113 | Sao_Nicolau | 65,0 | 17,92 | 10,10 | 7,19 | 8,25 | M |
| Passer iagoensis | SNI31 | N16º34.488 | W24º17.113 | Sao_Nicolau | 65,0 | 18,95 | 10,22 | 7,38 | 8,25 | M |
| Passer iagoensis | SNI32 | N16º34.488 | W24º17.113 | Sao_Nicolau | 66,0 | 18,59 | 9,90 | 7,19 | 8,25 | M |
| Passer iagoensis | SNI33 | N16º34.488 | W24º17.113 | Sao_Nicolau | 66,0 | 18,54 | 10,06 | 7,44 | 8,31 | M |
| Passer iagoensis | SNI34 | N16º34.488 | W24º17.113 | Sao_Nicolau | 65,0 | 18,33 | 10,06 | 7,32 | 8,45 | M |
| Passer iagoensis | SNI35 | N16º34.488 | W24º17.113 | Sao_Nicolau | 63,0 | 17,54 | 10,39 | 7,15 | 8,18 | M |
| Passer iagoensis | SNI36 | N16º34.488 | W24º17.113 | Sao_Nicolau | 64,0 | 18,15 | 10,03 | 7,28 | 8,30 | M |
| Passer iagoensis | SNI37 | N16º34.488 | W24º17.113 | Sao_Nicolau | 66,0 | 18,44 | 10,24 | 6,82 | 8,06 | M |
| Passer iagoensis | SV01 | N16°51.674 | W24°56.261 | Sao_Vicente | 66,0 | 17,91 | 9,56 | 5,86 | 7,78 | M |
| Passer iagoensis | SV02 | N16°51.674 | W24°56.261 | Sao_Vicente | 65,0 | 18,88 | 10,36 | 6,29 | 8,40 | M |
| Passer iagoensis | SV03 | N16°51.674 | W24°56.261 | Sao_Vicente | 66,5 | 19,10 | 9,28 | 7,15 | 8,16 | M |
| Passer iagoensis | SV04 | N16°51.674 | W24°56.261 | Sao_Vicente | 66,0 | 18,27 | 9,12 | 6,59 | 7,91 | M |
| Passer iagoensis | SV05 | N16°51.674 | W24°56.261 | Sao_Vicente | 68,5 | 18,74 | 9,68 | 6,98 | 8,23 | M |
| Passer iagoensis | SV06 | N16°51.674 | W24°56.261 | Sao_Vicente | 66,0 | 19,14 | 9,94 | 6,53 | 8,41 | M |
| Passer iagoensis | SV07 | N16°51.674 | W24°56.261 | Sao_Vicente | 64,0 | 18,17 | 8,97 | 6,92 | 7,89 | M |
| Passer iagoensis | SV10 | N16°51.674 | W24°56.261 | Sao_Vicente | 66,0 | 19,01 | 9,78 | 7,22 | 8,03 | M |
| Passer iagoensis | SV15 | N16°51.674 | W24°56.261 | Sao_Vicente | 66,0 | 17,72 | 9,08 | 6,71 | 8,02 | M |
| Passer iagoensis | SV16 | N16°51.674 | W24°56.261 | Sao_Vicente | 66,0 | 19,33 | 9,76 | 7,24 | 7,92 | M |
| Passer iagoensis | SV17 | N16°51.674 | W24°56.261 | Sao_Vicente | 67,0 | 18,75 | 9,77 | 6,96 | 7,75 | M |
| Passer iagoensis | SV18 | N16°51.674 | W24°56.261 | Sao_Vicente | 66,0 | 18,76 | 9,71 | 6,74 | 8,06 | M |
| Passer iagoensis | SV19 | N16°51.674 | W24°56.261 | Sao_Vicente | 68,0 | 18,32 | 10,63 | 6,88 | 7,84 | M |
| Passer iagoensis | SV20 | N16°51.674 | W24°56.261 | Sao_Vicente | 65,5 | 18,15 | 9,47 | 6,86 | 7,84 | M |
